# Valorization of *Nigella sativa* Seed Meal and Whey as Functional Ingredients to Enhance Nutritional, Rheological and Sensory Properties of Rice-Based Gluten-Free Bread

**DOI:** 10.3390/foods15132258

**Published:** 2026-06-23

**Authors:** Ibtissem Sanah, Fairouz Djeghim, Muhammet Arici, Muhammed Ozgolet, Eylul Ozturk, Keltoum Babouche, Souad Cherak, Maria D’Elia, Luca Rastrelli

**Affiliations:** 1 Laboratoire de Génie Biologique Valorisation et Innovation des Produits Agroalimentaires, Institut ISTA-Ain M’Lila, Université Larbi Ben M’hidi Oum El-Bouaghi, Oum El-Bouaghi 04000, Algeria; 2 Laboratoire de Recherche en Sciences Alimentaires, Formulation, Innovation, Valorisation et Intelligence Artificielle (SAFIVIA), Institut de la Nutrition, de l’Alimentation et des Technologies Agro-Alimentaires (INATAA), Université Frères Mentouri Constantine 1, Constantine 25017, Algeria; keltoum.babouche@doc.umc.edu.dz; 3 Équipe FNPAA, Laboratoire de Nutrition et Technologie Alimentaire (L.N.T.A), Institut de la Nutrition, de l’Alimentation et des Technologies Agro-Alimentaires (INATAA), Université Frères Mentouri Constantine 1, Constantine 25017, Algeria; fairouze.djeghim@umc.edu.dz; 4 Department of Food Engineering, Faculty of Chemical and Metallurgical Engineering, Yildiz Technical University, Istanbul 34220, Türkiye; muarici@yildiz.edu.tr (M.A.); mozgolet@yildiz.edu.tr (M.O.); eylulmeltemozturk@gmail.com (E.O.); 5 Institut de la Nutrition, de l’Alimentation et des Te chnologies Agro-Alimentaires (INATAA), Université Frères Mentouri Constantine 1, Constantine 25017, Algeria; cheraksouade@gmail.com; 6 Department of Pharmacy, University of Salerno, Via Giovanni Paolo II, 132, 84084 Fisciano, Italy; mdelia@unisa.it; 7 National Biodiversity Future Center—NBFC, 90133 Palermo, Italy; 8 Dipartimento di Scienze della Terra e del Mare, University of Palermo, 90133 Palermo, Italy

**Keywords:** gluten-free bread, *Nigella sativa* seed meal, whey, functional ingredients, agro-industrial by-products, rheology, texture, nutritional enhancement

## Abstract

This study investigated the valorization of agro-industrial by-products, namely Nigella sativa seed meal (BCSM) and whey, as functional ingredients to improve the quality of rice-based gluten-free bread. A Response Surface Methodology (RSM) approach was applied to optimize formulation parameters and evaluate their effects on physicochemical, rheological, nutritional, and sensory properties. The optimized formulations showed distinct performance profiles depending on the rice matrix and ingredient balance. The optimized brown rice bread (OBRB), characterized by the highest BCSM incorporation (10 g), showed the most relevant functional and nutritional improvements, including increased dietary fiber, enhanced antioxidant activity, and reduced hardness and chewiness compared with the corresponding control. In contrast, the optimized red rice bread (ORRB), characterized by low BCSM content and higher whey incorporation, mainly contributed to improved specific volume and crumb structure. Rheological analysis revealed distinct structural behaviors, with BCSM contributing to a more rigid and structured matrix, while whey promoted a softer and more compliant dough system. Sensory evaluation confirmed that the incorporation of these by-products did not negatively affect acceptability, with overall acceptability scores ranging between 5 and 6. Overall, these results indicate that OBRB was the most promising formulation for functional enrichment, whereas ORRB was mainly associated with structural optimization. This study demonstrates that BCSM and whey can be strategically used as formulation-dependent ingredients for developing nutritionally enhanced and structurally improved gluten-free bread, contributing to the sustainable valorization of food industry by-products.

## 1. Introduction

The production of high-quality gluten-free bread remains a significant challenge in food technology [1], primarily due to the absence of the gluten network, which is responsible for structural integrity, elasticity, and gas retention within the dough [2,3]. In the absence of wheat flour, formulations typically rely on starches and gluten-free cereal flours, such as maize, potato, and rice. Among these, pigmented and whole grain rice (e.g., red and brown rice) flour has emerged as a particularly suitable ingredient for gluten-free production due to its favorable nutritional, functional and hypoallergenic properties [4,5,6,7]. However, such formulations generally result in loaves characterized by low specific volume, compact crumb structure, and reduced shelf-life stability [8].

To overcome these limitations, several studies have explored formulation optimization strategies, particularly through statistical approaches such as Response Surface Methodology (RSM) [9,10,11,12]. In this context, the incorporation of hydrocolloids, plant-derived ingredients, dietary fibers, and animal proteins (e.g., egg and dairy proteins) has been proposed to partially compensate for the lack of a gluten network and improve textural properties [3,9,13].

Black cumin (*Nigella sativa* L.) is a historically significant culinary and medicinal plant. The cold-pressed oil industry generates considerable quantities of press cake as a by-product, which is characterized by high levels of plant proteins, dietary fiber, and residual lipids [14]. In addition, this matrix is rich in bioactive compounds, making it a promising upcycled functional ingredient for gluten-free bakery applications [15,16,17].

Whey is a nutrient-rich co-product of cheese production, traditionally considered a waste stream in the dairy industry. Due to its content of essential amino acids, minerals, and bioactive compounds, whey represents a valuable ingredient for improving nutritional quality [18,19,20]. Moreover, whey proteins provide important techno-functional properties in baking, contributing to improved texture, volume, and overall product quality [21]. To the best of our knowledge, no previous study has simultaneously investigated both Black Cumin Seed Meal (BCSM) and whey protein enrichment in gluten-free bread formulations. Furthermore, the integration of these two distinctive functional by-products within an RSM optimization framework represents an entirely novel approach.

Therefore, the aim of the present study was to optimize the formulation of gluten-free bread based on rice flours, enriched with *Nigella sativa* seed meal and whey, using Response Surface Methodology (RSM). In addition, the study evaluated the physicochemical, rheological, nutritional, and sensory characteristics of the optimized formulations in comparison with a control sample.

## 2. Materials and Methods

### 2.1. Reagents and Solvents

All chemicals and reagents used in this study were of analytical grade and obtained from commercial suppliers. Specifically, 2,2-diphenyl-1-picrylhydrazyl (DPPH), Folin–Ciocalteu reagent, sodium carbonate, gallic acid, and ascorbic acid, together with analytical-grade ethanol and methanol, as well as MS-grade acetonitrile, were purchased from Sigma-Aldrich (St. Louis, MO, USA). All reagents were used without further purification and stored according to the manufacturer’s instructions.

### 2.2. Bread-Making Materials

The composite flour was prepared by blending red rice flour (10.1% moisture, 1.8% ash, 2.4% fat, 7.4% protein, 75.5% carbohydrates, and 2.8% fiber), brown rice flour (9.9% moisture, 1.7% ash, 2.4% fat, 7.2% protein, 76.2% carbohydrates, and 2.6% fiber), corn flour (9.03% moisture, 1.77% ash, 1.7% fat, 8.1% protein, 77.2% carbohydrates, and 2.2% fiber), and tapioca starch (10.30% moisture, 0.25% ash, 0.27% fat, 0.5% protein, 88.08% carbohydrates, and 0.6% fiber).

All flours were procured from the local Algerian market (NaturAlimn, Bordj El Kifan, Algeria). Additional ingredients included salt (ENAsel, Sétif, Algeria) and instant dry yeast (Saf-Instant, Maisons-Alfort, France), which were also obtained from the local market.

### 2.3. By-Product Materials

The raw materials used in this study consisted of by-products obtained from two Algerian food industries. Black cumin seed meal (BCSM) was collected as a residue from cold mechanical oil extraction performed by El Mouna Company (Sdira No. 01, Ain Smara, Constantine, Algeria). The composition of the meal was as follows: 2.71% moisture, 8.50% ash, 6.90% fat, 22.18% protein, 58.33% carbohydrates, and 1.38% fiber.

Whey powder was supplied by Torche Dairy (Ouled Hamla, Oum El Bouaghi, Algeria) and was characterized by 68.2% lactose, 3.5% moisture, 15% ash, 1.1% fat, and 12.2% protein. All by-product powders were stored at 4 °C until further use.

### 2.4. Characterization of Phenolic Compounds of BCSM by HPLC Analysis

Phytochemical extraction was performed using a solvent-based protocol. Briefly, 20 g of Black Cumin Seed Meal (BCSM) powder were homogenized with 200 mL of a methanol-water mixture (70:30, *v*/*v*). The suspension was continuously stirred for 8 h at 25 °C to facilitate the maximum recovery of phenolic fractions. The resulting extract was filtered through a 0.45 μm membrane, and the solvent was subsequently eliminated under reduced pressure at 40 °C using a rotary evaporator.

Next, BCSM extracts were dissolved in HPLC-grade methanol, centrifuged, and subsequently filtered through a 0.22 µm membrane filter. Chromatographic analyses were performed using an Agilent 1200 HPLC system equipped with a photodiode array detector, quaternary pump, autosampler, and column oven.

Phenolic compounds, including gallic acid, protocatechuic acid, catechin, 4-hydroxybenzoic acid, syringic acid, ellagic acid, *m*-coumaric acid, *o*-coumaric acid, chrysin, caffeic acid, *p*-coumaric acid, ferulic acid, myricetin, quercetin, and kaempferol, were separated on a Waters Atlantis C18 column (250 mm × 4.6 mm, 5 μm).

A linear gradient elution was applied using a mobile phase consisting of solvent A (acetic acid/water, 0.1:99.9, *v*/*v*) and solvent B (acetic acid/acetonitrile, 0.1:99.9, *v*/*v*), at a flow rate of 1 mL/min. The gradient program was as follows: 10–60% B (0–15 min), 60% B (15–20 min), 60–10% B (20–25 min), and 10% B (25–30 min).

Chromatograms were recorded at 278, 320, and 360 nm, with spectral acquisition in the range of 190–400 nm.

### 2.5. Bread-Making Process

#### 2.5.1. Pre-Hydration of By-Products

In accordance with the findings of [22,23], the pre-hydration of by-products was applied to reduce the amount of additional water required during dough preparation and to minimize competitive water absorption between the dough matrix and dehydrated ingredients.

In this study, by-product powders were rehydrated using a solid-to-liquid ratio (*w*/*v*) by immersion in distilled water and left to equilibrate overnight (12 h) at ambient temperature (25 °C). The amount of water used for pre-hydrating the BCSM and whey was determined based on the water holding capacity of the by-products, which was found to be (32 mL water/10 g of BCSM and 1 mL water/1 g of whey).

#### 2.5.2. Bread Preparation

The formulations used for the preparation of the 40 gluten-free bread samples are presented in Table 1. Each formulation was based on 100 g of flour composed of two different rice varieties. Both formulations were supplemented with corn flour at a 1:1 (*w*/*w*) ratio and enriched with black cumin seed meal (BCSM) and whey (Table 1). All by-product powders were pre-hydrated prior to incorporation.

The bread-making process consisted of mixing flour, yeast, by-products, and water for 1 min at medium speed using a mixer (Moulinex OW240E30, Écully, France). Subsequently, salt previously dissolved in water was gradually added to the mixture at medium speed. The dough was then mixed for an additional 6 min, divided, molded, and placed into rectangular cardboard baking molds (9 × 7 × 3 cm).

A single-stage fermentation was performed in the baking trays to minimize degassing of the gluten-free matrix in a controlled fermentation chamber (Model HCP108, Memmert GmbH + Co. KG, Schwabach, Germany) at 37 °C for 30 min and 85% relative humidity. Baking was performed in an electric oven (Memmert D39263/D39264, Schwabach, Germany) at 230 °C for 30 min. After baking, the loaves were cooled to room temperature prior to further analysis.

The control bread consisted of the basic rice–corn flour formulation containing yeast, salt, and water, but without the addition of Black Cumin Seed Meal (BCSM) or whey protein.

### 2.6. Experimental Design

Response Surface Methodology (RSM) was employed to optimize the formulation of gluten-free bread. The experimental design considered four independent variables: water hydration level (X_1_: 70–100 mL), whey content (X_2_: 0–5.02 g), black cumin seed meal (BCSM) concentration (X_3_: 0.4–10 g), and rice variety (X_4_: Red Rice [RR] or Brown Rice [BR]) (Table 1). The selection of these variables and their respective ranges was based on preliminary trials and established experimental design principles.

The effects of these four independent variables (X_1_–X_4_) on the physicochemical and textural properties of bread were evaluated. The response variables included specific volume (Y_1_), moisture content (Y_2_), hardness (Y_3_), chewiness (Y_4_), and springiness (Y_5_). A Central Composite Design (CCD) was applied to the three quantitative factors (water, whey, and BCSM) at five coded levels (−α, −1, 0, +1, +α; α = 1.68). Rice variety (red rice or brown rice) was included as a categorical factor evaluated at two levels, and the CCD matrix was replicated for each rice variety. The experimental design comprised 40 runs, including 12 replicates at the center point (0,0,0) for each rice variety to ensure reproducibility.

To validate the optimal conditions predicted by the model, confirmatory experiments were conducted using the optimized factor levels. A second-order polynomial equation (Equation (1)) was used to describe the relationship between the response variables and the independent factors:

Y = b_0_ + b_1_X_1_ + b_2_X_2_ + b_3_X_3_ + b_11_X_1_^2^ + b_22_X_2_^2^ + b_33_X_3_^2^ + b_12_X_1_X_2_ + b_13_X_1_X_3_ + b_23_X_2_X_3_ (1)

where Y is the predicted response; b_0_ is the intercept; b_1_–b_3_ are the linear coefficients; b_11_–b_33_ are the quadratic coefficients; and b_12_–b_23_ are the interaction coefficients. X_1_, X_2_, and X_3_ represent the continuous formulation factors. Rice variety (X_4_) was treated as a categorical blocking factor; therefore, it was used for comparative statistical analysis between red rice and brown rice matrices but was not included as a continuous polynomial term in the predictive equations.

#### 2.6.1. Determination of Specific Volume (Vsp)

Bread weight and volume were measured 1 h after baking. The specific volume of all formulations (*n* = 40), including control and enriched samples, was determined using a displacement method according to AACC [24] guidelines. Specific volume (cm^3^/g) was calculated as the ratio of bread volume to mass.

#### 2.6.2. Determination of Moisture Content

Moisture content was determined in triplicate following AOAC method 934.06 [25]. Samples were dried in an oven (Memmert GmbH, Schwabach, Germany) at 105 °C until a constant weight was achieved.

#### 2.6.3. Texture Profile Analysis (TPA)

Texture profile analysis (TPA) was performed using a TA HD Plus Texture Analyzer (Stable Micro Systems, Godalming, UK) equipped with a 5 kg load cell and a 36 mm cylindrical probe. Bread samples (2 × 2 × 2 cm cubes) were analyzed 2 h after baking to ensure complete cooling.

A two-cycle compression test was applied under the following conditions: test speed, 1.7 mm/s; compression, 30%; and time between cycles, 5 s. The parameters determined from the TPA curves were hardness, springiness, and chewiness [26]. The compression level (30%) was selected according to standard bread TPA protocols reported in the literature and preliminary trials, allowing reliable characterization of crumb mechanical properties while avoiding complete structural collapse. Measurements were performed in triplicate for each formulation.

### 2.7. Comparison of Control and Optimized Gluten-Free Bread

Optimized and control gluten-free bread samples were characterized one hour after baking, while rheological measurements of the dough were performed prior to baking. Physical evaluations included baking loss, specific volume, pH, crumb cellular structure, and water activity (aw). In addition, textural and chromatic properties (color) were assessed, together with sensory evaluation. Furthermore, proximate composition (moisture, protein, lipids, ash, fiber, and carbohydrates) was determined, along with total phenolic content and antioxidant capacity.

For rheological analysis, fortified gluten-free doughs were prepared by incorporating by-products at different levels, following the same bread-making procedure described above. The viscoelastic properties of the dough were measured at 25 °C using a rheometer (MCR 302; Anton Paar, Sydney, NSW, Australia) equipped with a Peltier temperature control system and parallel plate geometry (PP25).

To determine the linear viscoelastic region, an amplitude sweep test was conducted over a strain range of 0.01–100%. Frequency sweep measurements were then performed in the range of 0.1–10 Hz (0–100 rad/s) at a constant strain of 0.03%. The gap was set to 1 mm during all measurements. After positioning the sample, a resting time of 3 min was allowed to ensure relaxation of residual stresses. The number of measuring points was 11.

The storage modulus (G′), loss modulus (G″), and damping factor were recorded as a function of frequency.

#### 2.7.1. Weight Loss

The baking loss was calculated by weighing the bread dough prior to baking and the finished product an hour after baking, expressed as a percentage of the initial dough weight using the following Equation (2):

(2) $$\%weight\;loss\left(WL\right)=\frac{Dough\;weight-Bread\;weight}{Dough\;weight}\cdot 100\%$$

#### 2.7.2. pH Measurement

A 10 g sample of finely ground bread was combined with 100 mL of distilled water. The mixture was stirred magnetically for 30 min. Following a 10-min settling period, the pH of the resulting supernatant was measured with a penetration probe for semi-solid substances [27,28].

#### 2.7.3. Crust and Crumb Color of Bread

Color measurements were performed on both the crumb and crust of bread samples using a Hunter Lab colorimeter (Chroma Meter CR-400, Konica-Minolta Sensing Inc., Osaka, Japan). The color was quantified in the Lab* color space, where L* represents the lightness from black (0) to white (100), a* indicates the chromaticity on a green (negative) to red (positive) axis and b* indicates the chromaticity on a blue (negative) to yellow (positive) axis. A total of five samples were analyzed for each bread formulation, and the average Lab* values were calculated.

#### 2.7.4. Crumb Cell Analysis

The microstructure of gluten-free bread crumbs was analyzed using ImageJ software (version 1.43u; Java 1.7.0-2132 bit. Wayne Rasband. National Institutes of Health, Bethesda, MD, USA) as described by [29]. A digital image of each crumb was captured and saved in TIFF format. The bread was horizontally sliced into two pieces, and the resulting images were cropped to isolate the crumb structure. Each image was then converted to an 8-bit grayscale format to distinguish between the crumb pores (black) and the crumb matrix (white). The following parameters were measured: cell number per square millimeter, average pore size per square millimeter, pore area fraction (%), perimeter, solidity (0–1) and circularity (0–1).

#### 2.7.5. Water Activity

The water activity (aw) of the gluten-free bread samples was analyzed at 30 °C using a Novasina Lab Master aw meter (Novasina AG, Lachen, Switzerland).

#### 2.7.6. Proximate Composition

All proximate analyses were conducted on ground, dried bread samples and on by-products (*Nigella sativa* seed meal and whey), in accordance with standard methods [25].

Total protein content was determined using the Kjeldahl method (N × 6.25), following AOAC method 920.87. Total lipids were extracted by hot extraction using petroleum ether as the solvent, according to AOAC method 945.16 [25]. Ash content was determined by incineration at 550 °C, as described in AACC method 923.03 [25].

Crude fiber (CF) content was determined using the Weende method [30], based on acid–alkaline digestion with a Fibersac^®^ automated system. CF was calculated as the difference between the dried residue and the incinerated ash and expressed as a percentage of the initial sample weight.

All analyses were performed in triplicate and expressed as mean ± standard deviation (SD). Total carbohydrate content was calculated by difference according to Equation (3):

(3) Carbohydrates (%)=100−(%moisture+%protein+%ash+%fat+%fiber)

Carbohydrate content was expressed as g/100 g of sample. Total energy (kcal/100 g) was calculated using conversion factors of 9 kcal/g for lipids and 4 kcal/g for both proteins and carbohydrates [31,32].

#### 2.7.7. Determination of Total Phenolic Content and Antioxidant Activity of Bread

Bread extracts for the determination of total phenolic content and antioxidant activity were prepared following a slightly modified method of [33]. Briefly, dried bread samples were ground into a fine powder, and a 0.5 g aliquot was extracted with 10 mL of 80% (*v*/*v*) methanol. The mixture was subjected to magnetic stirring for 2 h, followed by centrifugation at 2000 rpm for 15 min at 4 °C (Hettich Universal 320R centrifuge, Andreas Hettich GmbH & Co. KG, Tuttlingen, Germany).

The resulting supernatant was filtered and collected for further analysis. All extractions were performed in triplicate to ensure reproducibility.

The total phenolic content (TPC) of the enriched bread samples was determined using the Folin–Ciocalteu colorimetric assay [33]. Briefly, 0.3 mL of extract was mixed with 1.5 mL of diluted Folin–Ciocalteu reagent (1:10, *v*/*v*) and 1.2 mL of sodium carbonate solution (6%, *w*/*v*).

After incubation for 90 min in the dark at room temperature, absorbance was measured at 725 nm using a UV–Vis spectrophotometer (Jenway 7305, Bibby Scientific Ltd., Stone, Staffordshire, UK), with distilled water as the blank. All analyses were performed in duplicate.

Results were expressed as milligrams of gallic acid equivalents per gram of dry extract (mg GAE/g).

The total antioxidant activity of the bread extracts was determined using the DPPH radical scavenging assay, following the method described by [34] with slight modifications. Briefly, 100 µL of each extract was mixed with 4 mL of 80% (*v*/*v*) methanol and 1900 µL of a freshly prepared DPPH solution (1 mmol/L), resulting in a final DPPH concentration of 0.167 mmol/L.

The reaction mixture was incubated in the dark for 30 min at room temperature. Absorbance was then measured at 517 nm using a UV–Vis spectrophotometer (Jenway 7305, Bibby Scientific Ltd., Stone, Staffordshire, UK). Ascorbic acid was used as the reference standard for calibration.

A 0.11 mM DPPH methanolic solution was prepared by dissolving 3.4 mg of DPPH in 100 mL of methanol. Radical scavenging activity was expressed as the percentage inhibition (PI), calculated using Equation (4):

(4) $$\mathrm{PI}\;(\%)=\frac{A_{\mathrm{control}}-A_{\mathrm{test}}}{A_{\mathrm{control}}}\times 100$$

where $A_{\mathrm{control}}$ is the absorbance of the control (without sample) and $A_{\mathrm{test}}$ is the absorbance of the sample.

### 2.8. Sensory Analysis

Following the recommendations of Watts et al. [35], a trained sensory panel consisting of 10 members (5 women and 5 men) with an age range of [25 to 45] years from Larbi Ben M’hidi University (Oum El-Bouaghi, Algeria) was established to evaluate the sensory quality of optimized gluten-free bread samples in comparison with control samples.

The panel was composed of academic staff and postgraduate students who were regular bread consumers. Panelists were trained prior to evaluation to ensure consistency and reliability of the results.

Bread samples were prepared by cutting each loaf into 10 equal portions (approximately 5 g each). During each session, panelists evaluated two randomized samples, identified by three-digit codes, 2 h after baking.

Seven sensory attributes were assessed: shape, taste, color, odor, texture/mouthfeel, alveolation, and overall acceptability. Water was provided for palate cleansing between samples.

Sensory attributes were evaluated using a 10 cm unstructured line scale, and scores were subsequently converted into a 10-point numerical scale.

In addition, a paired preference test was conducted to determine statistically significant differences between formulations. Panelists were asked to select their preferred sample based on overall sensory perception [36,37].

### 2.9. Statistical Analysis

All analytical experiments were performed in triplicate, and the experimental data are expressed as mean ± standard deviation (SD). A Central Composite Design (CCD) framework was applied to evaluate the continuous formulation factors. The experimental data were fitted to second-order polynomial models using regression analysis.

To accommodate both continuous inputs (X_1_, X_2_, X_3_) and the qualitative factor (X_4_, rice variety), an Analysis of Covariance (ANCOVA) framework was applied to the mixed models using the Ordinary Least Squares (OLS) method. The significance of linear, quadratic, and interaction terms was evaluated by Analysis of Variance (ANOVA) at a confidence level of *p* < 0.05.

Since rice variety (X_4_) was treated as a categorical blocking factor, it was not included as a continuous numerical term in the final predictive equations. X_4_-related effects and interactions were evaluated in the statistical model to compare the two rice matrices, whereas the final predictive equations were expressed separately for each rice variety using only the continuous formulation factors.

Model adequacy was verified using the coefficient of determination (R^2^), adjusted R^2^, and lack-of-fit testing. To preserve model hierarchy and ensure consistency between the fitted models, response surface plots, optimization procedure, and predictive equations, complete quadratic models including all linear, quadratic, and interaction terms were retained throughout the RSM analysis, regardless of the statistical significance of individual coefficients. Although significant lack of fit was observed for some responses, the models were retained because they remained highly significant (*p* < 0.0001) and exhibited satisfactory coefficients of determination and adjusted R^2^ values. The observed lack of fit was mainly attributed to the extremely low variability observed among replicated center-point runs, which generated a very small pure error and increased the sensitivity of the lack-of-fit test. Therefore, despite the observed lack of fit, the models were considered adequate for identifying formulation trends and optimization regions within the experimental domain.

For the direct comparison of means among multiple distinct samples, one-way ANOVA followed by Tukey’s Honestly Significant Difference (HSD) post hoc test was employed.

To validate the optimization process, Student’s *t*-test was conducted to assess significant differences between the independent control bread and the optimized formulations. In addition, a one-sample *t*-test was conducted to compare the experimentally observed mean of the optimized bread against the fixed theoretical values predicted by the RSM models.

All statistical modeling, matrix designs, and optimization procedures were performed using JMP Pro software, version 17.0.0 (SAS Institute Inc., Cary, NC, USA).

## 3. Results and Discussion

### 3.1. HPLC Profile of Phenolic Compounds

**Figure 1:**
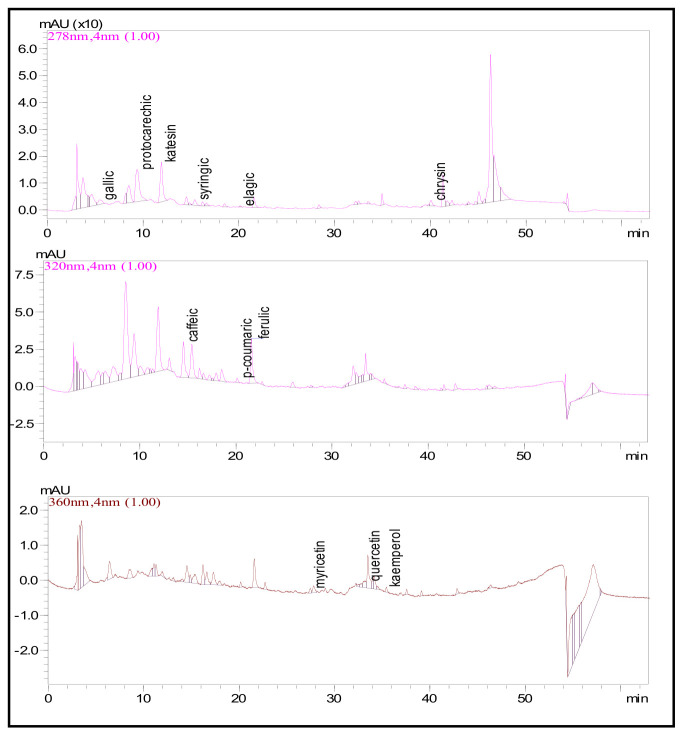
Chromatogram of phenolic compounds of black cumin identified at 278, 320 and 360 nm by HPLC analysis.

The chromatographic profiles obtained by HPLC analysis at 278, 320, and 360 nm (Figure 1) revealed a complex and diverse phenolic composition of the black cumin seed meal (BCSM), confirming its potential as a functional ingredient in gluten-free formulations. Detection at 278 nm highlighted the presence of low-molecular-weight phenolic acids and flavan-3-ols, including gallic acid, protocatechuic acid, catechin, syringic acid, ellagic acid, and chrysin. Among these, a prominent peak observed at approximately 47 min suggests the presence of a major compound, likely contributing significantly to the antioxidant potential of the matrix. At 320 nm, the chromatographic profile was dominated by hydroxycinnamic acids, such as caffeic acid, *p*-coumaric acid, and ferulic acid, which are known for their strong antioxidant and antimicrobial properties. These compounds are particularly relevant in bakery applications, as they may contribute to oxidative stability and shelf-life extension of gluten-free products. At 360 nm, the chromatograms confirmed the presence of flavonols, including myricetin, quercetin, and kaempferol, which eluted at longer retention times (25–35 min), consistent with their higher molecular complexity. These compounds are widely recognized for their bioactive properties, including antioxidant, anti-inflammatory, and potential health-promoting effects. Overall, the HPLC profile demonstrates that BCSM is a rich source of phenolic compounds with different chemical structures and functionalities. This compositional diversity is likely to play a key role in enhancing both the nutritional value and the functional performance of gluten-free bread formulations developed in this study.

### 3.2. Influence of Independent Variables on the Quality of Gluten-Free Bread

**Figure 2:**
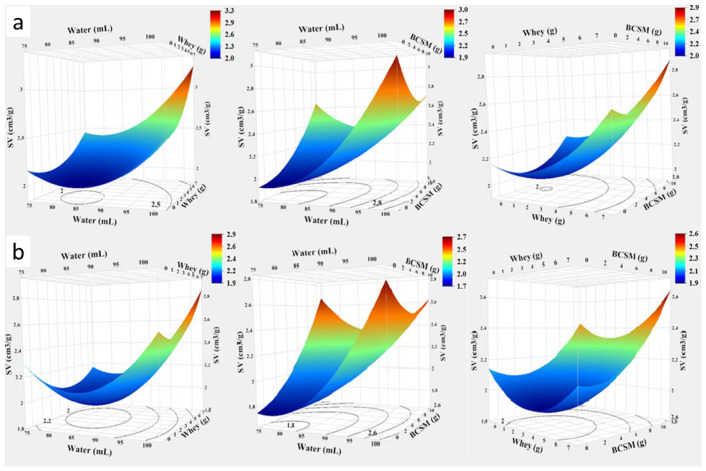
Three-dimensional response surface plots showing the effect water, whey, BCSM and rice variety ((**a**): red rice; (**b**): brown rice) on gluten-free bread specific volume.

**Figure 3:**
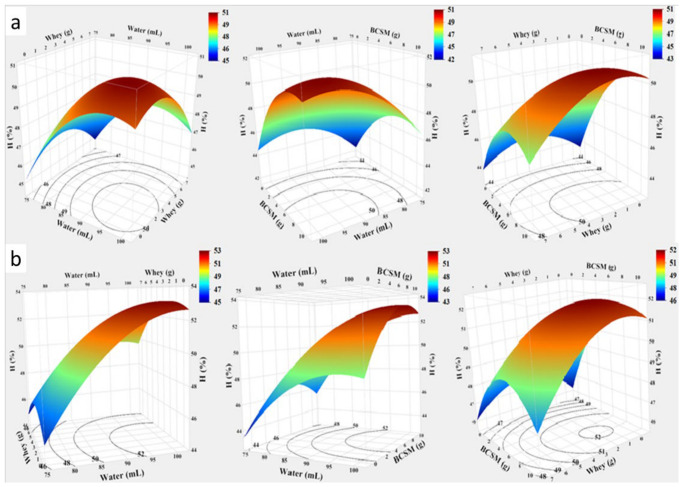
Three-dimensional response surface plots showing the effect water, whey, BCSM and rice variety ((**a**): red rice; (**b**): brown rice) on gluten-free bread moisture.

**Figure 4:**
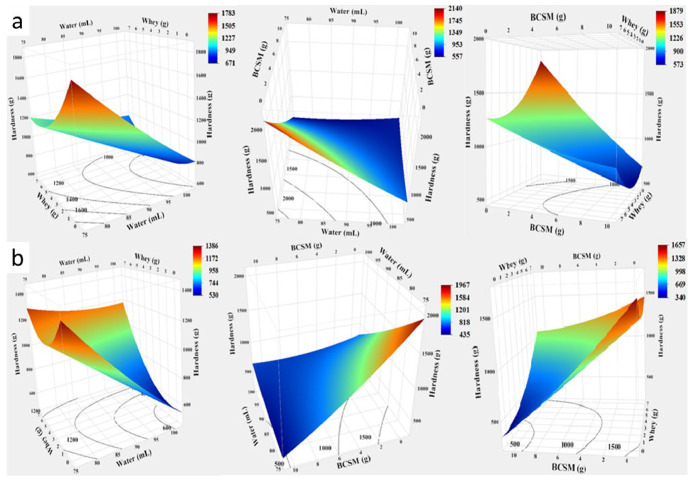
Three-dimensional response surface plots showing the effect water, whey, BCSM and rice variety ((**a**): red rice; (**b**): brown rice) on gluten-free bread hardness.

**Figure 5:**
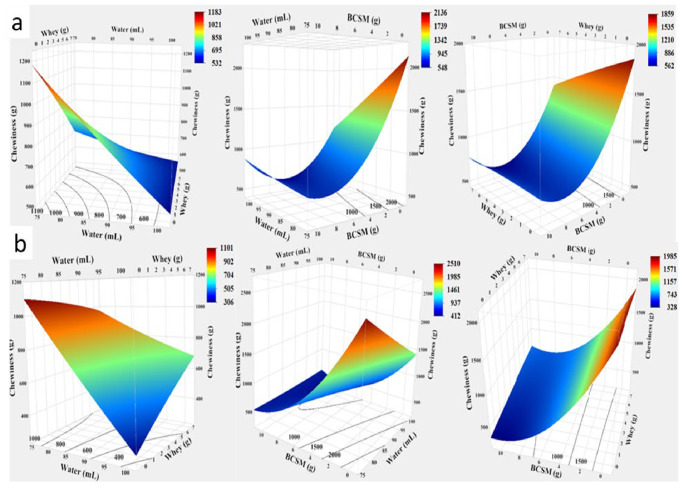
Three-dimensional response surface plots showing the effect water, whey, BCSM and rice variety ((**a**): red rice; (**b**): brown rice) on gluten-free bread chewiness.

**Figure 6:**
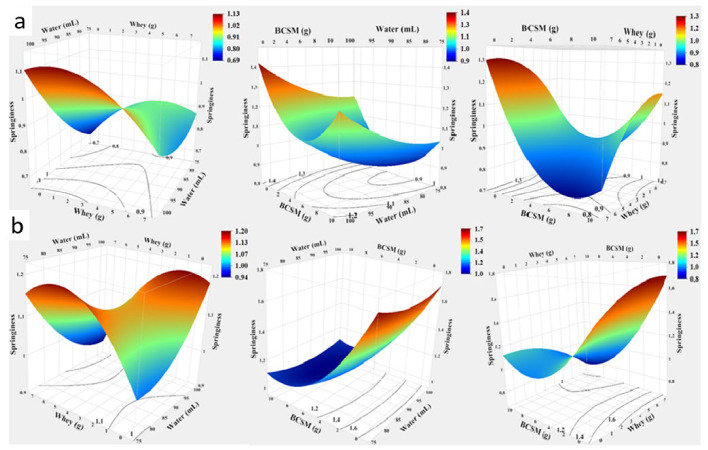
Three-dimensional response surface plots showing the effect water, whey, BCSM and rice variety ((**a**): red rice; (**b**): brown rice) on gluten-free bread springiness.

Because rice variety was treated as a categorical factor, separate response surfaces were generated for red rice and brown rice formulations using the fitted continuous-factor models. Therefore, the following discussion highlights both the individual behavior of each rice matrix and the comparative differences between them, in order to identify the most suitable gluten-free bread formulation. The effects of water content, whey, BCSM, and rice variety on the quality attributes of the 40 gluten-free bread formulations are illustrated in Figure 2, Figure 3, Figure 4, Figure 5 and Figure 6 and summarized in Table 2.

#### 3.2.1. Effect of Independent Variables on Specific Volume

The specific volume (Vsp) of the gluten-free bread samples varied significantly (*p* < 0.0001) across the 40 experimental trials, ranging from 1.84 to 2.58 cm^3^/g (Table 2).

Regression analysis revealed that water content (X_1_) had the most pronounced influence on specific volume, highlighting its critical role in dough expansion and gas retention. Whey (X_2_) and BCSM (X_3_) also significantly affected Vsp, confirming their contribution to the structural development of gluten-free bread. The significance of quadratic terms (*p* < 0.05) indicates a non-linear behavior of the system, as confirmed by the response surface plots (Figure 2). In particular, a significant antagonistic interaction between water and BCSM (b_13_ = −0.062, *p* = 0.026) was observed, suggesting that excessive levels of both components may negatively affect expansion, likely due to increased dough density or competition for water. Conversely, the interaction between whey and rice variety (b_24_ = 0.0528, *p* = 0.015) showed a synergistic effect, indicating that the type of rice (especially red rice) modulates the functional performance of whey proteins in improving crumb structure. Although X_4_ was not included in the final polynomial predictive equations, its interaction effects were evaluated within the ANCOVA mixed-model framework. These findings demonstrate that the optimization of water content in combination with functional ingredients is essential to maximize specific volume in gluten-free bread formulations. The regression model exhibited a satisfactory goodness of fit, with a coefficient of determination (R^2^) of 81.92%, indicating that the model adequately explains the variability of the response. The predictive equations for specific volume for red rice (RR) and brown rice (BR) are reported below (Equations (5) and (6)):

(5) $$\begin{array}{c} Y_{1,RR}=12.02-0.2533X_{1}-0.205X_{2}+0.233X_{3}+0.001594X_{1}^{2}+0.01109X_{2}^{2}+0.00493X_{3}^{2}+0.00200X_{1}X_{2}\\ -0.00309X_{1}X_{3}+0.00237X_{2}X_{3} \end{array}$$

(6) $$\begin{array}{c} Y_{1,BR}=12.34-0.2573X_{1}-0.256X_{2}+0.250X_{3}+0.001594X_{1}^{2}+0.01109X_{2}^{2}+0.00493X_{3}^{2}+0.00200X_{1}X_{2}\\ -0.00309X_{1}X_{3}+0.00237X_{2}X_{3} \end{array}$$

#### 3.2.2. Effect of Independent Variables on Moisture Content

The moisture content of the gluten-free bread samples ranged from 46.16% to 53.09%, remaining within acceptable limits for gluten-free bakery products (Table 2). A second-order polynomial regression model was applied to evaluate the effects of the independent variables (X_1_, X_2_, X_3_, and X_4_) on moisture content (Table 3). Table 3 reports the statistical effects estimated from the mixed model, including the categorical factor X_4_ and its interactions with the continuous variables. However, since X_4_ represents rice variety and was treated as a categorical blocking factor, the final predictive equations were expressed separately for red rice and brown rice and therefore include only the continuous variables X_1_, X_2_, and X_3_. The analysis revealed that all linear factors, water content (X_1_), whey (X_2_), BCSM (X_3_), and rice variety (X_4_), had a statistically significant effect on moisture (*p* < 0.05), highlighting their key role in determining water retention in the final product. The negative coefficients of the quadratic terms (X_1_^2^, X_2_^2^, X_3_^2^) indicate a parabolic relationship, suggesting that excessive levels of these components may lead to a decrease in moisture content beyond an optimal point. This behavior is typical of multicomponent systems where water binding and redistribution phenomena become limiting at higher concentrations. Most interaction terms (X_1_X_2_, X_1_X_3_, X_2_X_3_, X_2_X_4_, X_3_X_4_) were not statistically significant (*p* > 0.05), indicating that these combined effects did not substantially influence moisture within the studied range. However, the interaction between water content and rice variety (b_14_ = −0.331; *p* = 0.051) was close to the significance threshold, suggesting a potential modulatory effect of the rice matrix on water absorption and retention.

The intercept (b_0_ = 50.82) represents the predicted moisture content at the central levels of all variables. Overall, the model indicates that linear effects dominate moisture behavior, while quadratic terms refine the prediction by accounting for curvature in the response surface (Figure 3). The coefficient of determination (R^2^ = 86.14%) confirms the good predictive ability of the model, indicating that it adequately explains the variability in moisture content and can be reliably used for formulation optimization. The regression equations describing moisture content for red rice (RR) and brown rice (BR) are presented below (Equations (7) and (8)):

(7) $$\begin{array}{c} Y_{2,RR}=-41.7+1.844X_{1}+1.55X_{2}+0.555X_{3}-0.00974X_{1}^{2}-0.0908X_{2}^{2}-0.0635X_{3}^{2}-0.0103X_{1}X_{2}\\ +0.0071X_{1}X_{3}-0.0346X_{2}X_{3} \end{array}$$

(8) $$\begin{array}{c} Y_{2,BR}=-47.8+1.941X_{1}+1.50X_{2}+0.398X_{3}-0.00974X_{1}^{2}-0.0908X_{2}^{2}-0.0635X_{3}^{2}-0.0103X_{1}X_{2}\\ +0.0071X_{1}X_{3}-0.0346X_{2}X_{3} \end{array}$$

#### 3.2.3. Effect of Independent Variables on Hardness

The hardness of the gluten-free bread samples showed a wide variation, ranging from 428.67 g to 1708.99 g (Table 2), indicating a strong influence of formulation variables on crumb structure. Regression analysis revealed that water content (X_1_) and BCSM (X_3_) were the main factors affecting hardness, both exhibiting negative linear coefficients (b_1_ = −133.5 and b_3_ = −216.2). This indicates that increasing these components significantly reduces hardness, leading to a softer and less compact crumb structure. The softening effect of water is associated with increased plasticization of the dough matrix, whereas BCSM likely contributes through fiber-induced weakening of the starch network and improved moisture retention. The interaction between water and BCSM (X_1_X_3_) showed a positive coefficient (b_13_ = 83.5), suggesting that at higher combined levels, the softening effect is partially attenuated. This behavior may be attributed to the formation of complex interactions between proteins, fibers, and phenolic compounds, which can reinforce the matrix and limit excessive softening. Whey (X_2_) and rice variety (X_4_) exhibited smaller linear effects on hardness (b_2_ = 5.1 and b_4_ = 46.0), indicating a secondary contribution to texture. However, their quadratic (X_2_^2^ = 51.9) and interaction terms (e.g., X_2_X_4_ = −60.3) highlight a non-linear behavior, suggesting that their effect depends on concentration and interactions with other formulation components. The response surface plots (Figure 4) revealed a characteristic saddle-shaped profile, indicating the presence of both minima and maxima within the experimental domain. This confirms that hardness is governed by complex interactions among variables rather than simple linear relationships. The observed U-shaped curvature further suggests that, beyond certain levels, the addition of ingredients may lead to a reversal of the softening effect and a subsequent increase in firmness. The model explained 84.89% of the variability in hardness (R^2^ = 84.89%), indicating good predictive capability. The regression equations for hardness for red rice (RR) and brown rice (BR) are presented below (Equations (9) and (10)):

(9) $$Y_{3,RR}=8157-91.4X_{1}-431X_{2}-490X_{3}+0.216X_{1}^{2}+11.99X_{2}^{2}+2.06X_{3}^{2}+3.10X_{1}X_{2}+4.11X_{1}X_{3}+9.12X_{2}X_{3}$$

(10) $$Y_{3,BR}=7454-87.0X_{1}-373X_{2}-487X_{3}+0.216X_{1}^{2}+11.99X_{2}^{2}+2.06X_{3}^{2}+3.10X_{1}X_{2}+4.11X_{1}X_{3}+9.12X_{2}X_{3}$$

#### 3.2.4. Effect of Independent Variables on Chewiness

Chewiness values of the gluten-free bread samples ranged from 388.91 to 1770.79 (Table 2), indicating substantial variability in textural behavior as a function of formulation. Regression analysis identified BCSM (X_3_) and water content (X_1_) as the most influential factors, both exhibiting significant negative linear effects (b_3_ = −282.1; b_1_ = −115.7; *p* < 0.05). This indicates that increasing these components markedly reduces chewiness, resulting in a softer and less energy-demanding crumb during mastication. This behavior can be attributed to the plasticizing effect of water and the disruption of the starch-based network induced by the incorporation of fiber-rich BCSM. In contrast, whey (X_2_) and rice variety (X_4_) showed non-significant linear effects (*p* > 0.05), suggesting that their direct contribution to chewiness is limited under the investigated conditions (Table 3). A strong quadratic effect was observed for BCSM (b_33_ = 115.2; *p* < 0.001), indicating a non-linear relationship between BCSM concentration and chewiness. This suggests that while moderate levels of BCSM reduce chewiness, excessive incorporation may promote structural reorganization and partial recovery of firmness (Figure 5).

Regarding interaction effects, a significant positive interaction was found between water and BCSM (b_13_ = 68.9), as well as between BCSM and rice variety (b_34_ = 69.3). These results indicate that the combined presence of these variables partially counteracts their individual softening effects, likely due to enhanced matrix cohesion arising from interactions among fibers, proteins, and phenolic compounds. Conversely, a significant negative interaction between whey and rice variety (b_24_ = −43.6; *p* = 0.009) was observed, suggesting that their combined effect further reduces chewiness, possibly due to modifications in protein–starch interactions and water redistribution within the dough system. The response surface plots (Figure 5) confirmed the non-linear nature of the system, characterized by curved surfaces and localized optima, highlighting the importance of formulation balance in controlling chewiness. The regression model showed excellent predictive performance, with a coefficient of determination (R^2^ = 95.03%), indicating that the model explains nearly all variability in chewiness. The regression equations for chewiness for red rice (RR) and brown rice (BR) are reported below (Equations (11) and (12)):

(11) $$\begin{array}{c} Y_{4,RR}=5669-53.2X_{1}-237X_{2}-517.8X_{3}+0.081X_{1}^{2}-0.81X_{2}^{2}+13.03X_{3}^{2}+2.17X_{1}X_{2}+3.388X_{1}X_{3}+4.60X_{2}X_{3} \end{array}$$

(12) $$\begin{array}{c} Y_{4,BR}=6237-58.4X_{1}-195X_{2}-564.4X_{3}+0.081X_{1}^{2}-0.81X_{2}^{2}+13.03X_{3}^{2}+2.17X_{1}X_{2}+3.388X_{1}X_{3}+4.60X_{2}X_{3} \end{array}$$

#### 3.2.5. Effect of Independent Variables on Springiness

According to Table 2, springiness ranged from 0.86 to 1.68. Based on the regression coefficients provided in Table 3, the variables X_3_ (b_3_ = −0.0976) and X_4_ (b_4_ = −0.0912) exert a significant negative impact on springiness (*p* < 0.001). This indicates that increasing the levels of these two components leads to a substantial decrease in the elastic recovery of the matrix. The term X_3_^2^ is significant and positive (b_33_ = 0.0584, *p* = 0.006), suggesting a non-linear (curvilinear) relationship where the negative effect of X_3_ (BCSM) diminishes at higher concentrations, or perhaps initiates an upward trend (Figure 6). The interaction between X_3_ and X_4_ (X_3_X_4_) is statistically significant (b_34_ = 0.0550, *p* = 0.012). This confirms that the effect of X_3_ on springiness is strongly dependent on the level of X_4_ (rice variety), a classic indicator of a synergistic interaction in the food matrix. Variables X_1_ and X_2_ (water and whey) and their associated interactions (e.g., X_1_X_2_) show *p*-values > 0.05, indicating that these factors do not exert a statistically significant independent effect on springiness within the tested range. Equations (13) and (14) represent the regression model for springiness based on actual factors with R^2^ = 75.14%.

Y_5RR_ = 2.09 − 0.0383X_1_ + 0.222X_2_ − 0.059X_3_ + 0.000293X_1_X_1_ − 0.00594X_2_X_2_ + 0.00661X_3_X_3_ − 0.00172X_1_X_2_ + 0.00000X_1_X_3_ − 0.00632X_2_X_3_ (13)

Y_5BR_ = 3.20 − 0.0465X_1_ + 0.220X_2_ − 0.096X_3_ + 0.000293X_1_X_1_ − 0.00594X_2_X_2_ + 0.00661X_3_X_3_ − 0.00172X_1_X_2_ + 0.00000X_1_X_3_ − 0.00632X_2_X_3_ (14)

The statistical adequacy of the fitted polynomial models is summarized in Table 4. All fitted models were statistically significant (ANOVA *p* < 0.0001), with coefficients of determination (R^2^) ranging from 75.14% to 95.03%. Although significant lack of fit was observed for some responses, the models were retained because they showed high explanatory power and were used primarily to identify formulation trends and optimization regions within the experimental domain. The observed lack of fit is likely related to the very low pure error associated with the replicated center points, which increases the sensitivity of the lack-of-fit test.

As shown in Table 4, all fitted models were statistically significant (*p* < 0.05) and exhibited satisfactory coefficients of determination, supporting their suitability for describing the effects of formulation variables on bread quality attributes.

### 3.3. Optimization of Gluten-Free Bread Formulation

Response Surface Methodology (RSM) was applied to optimize the formulation of gluten-free bread, considering water content, whey, BCSM, and rice variety as independent variables. The optimization criteria were defined to maximize specific volume and springiness while minimizing hardness and chewiness, in order to achieve a desirable balance between structural integrity and textural acceptability. In addition, moisture content was constrained within a target range of 46–48% to ensure adequate product softness without compromising shelf-life stability. The optimized formulations obtained for both brown rice and red rice gluten-free breads, along with their corresponding predicted responses, are presented in Table 5. These optimized formulations were compared with the control samples (BRB-CTRL and RRB-CTRL), prepared without the addition of by-products, in order to evaluate the effectiveness of the proposed formulation strategy. The low BCSM level selected for ORRB should not be interpreted as a contradiction of the functional enrichment objective, but rather as the outcome of the multi-response optimization process. In the red rice matrix, higher BCSM levels negatively affected technological responses, particularly specific volume and crumb structure. Therefore, the model selected a low BCSM level combined with whey and high hydration to preserve structural quality. Conversely, the brown rice matrix tolerated and benefited from higher BCSM incorporation, leading to the selection of OBRB as the formulation most strongly oriented toward functional and antioxidant enrichment.

### 3.4. Experimental Validation of the Optimization Model

Table 6 summarizes the predicted and experimentally obtained responses under optimal conditions for both rice varieties, confirming the validity of the developed models. The experimental values for both optimized formulations (OBRB and ORRB) were in close agreement with the predicted values. Model validation was performed using a Student’s *t*-test, which revealed no significant differences between predicted and experimental responses (*p* > 0.05). This strong agreement confirms the adequacy and robustness of the optimization models applied in this study. A comparative analysis between the two optimized formulations highlights distinct technological behaviors. The optimized red rice bread (ORRB) exhibited a higher specific volume (2.39 cm^3^/g) compared to the optimized brown rice bread (OBRB) (2.21 cm^3^/g), suggesting improved gas retention capacity and a more extensible dough matrix. This effect can be attributed to the higher water content and the presence of whey proteins, which enhance dough viscoelasticity and expansion during baking. In contrast, OBRB showed significantly lower hardness (546.35 g) and chewiness (546.35) compared to ORRB (912.53 g and 950.66, respectively), indicating a softer crumb structure. This behavior is likely associated with the higher incorporation level of BCSM (10%), which contributes to increased water retention and disruption of the starch network through its fiber content. Both formulations exhibited high springiness values (>1.0), although ORRB demonstrated slightly higher elasticity (1.32), suggesting a more cohesive and resilient crumb structure.

Overall, these results indicate that while both formulations were successfully optimized, ORRB tends to produce a more structured and elastic crumb, whereas OBRB yields a softer and less resistant texture. From a technological perspective, the latter may be advantageous in gluten-free products, where softness is often associated with improved consumer acceptability and similarity to wheat-based bread.

**Figure 7:**
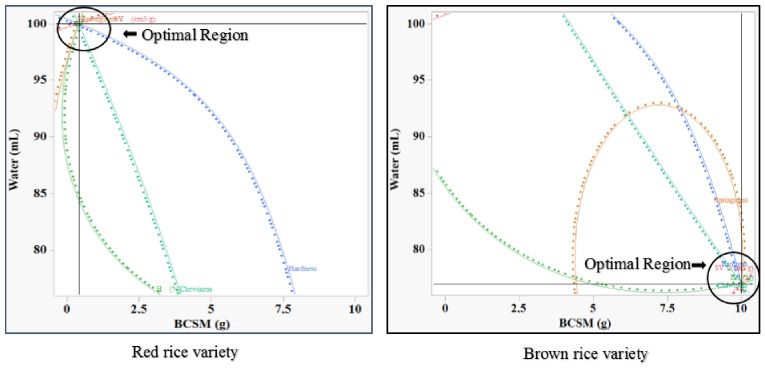
Overlaid contour plots illustrating the optimal region for water content and BCSM levels that satisfy multiple response constraints (hardness, springiness, chewiness, moisture content, and specific volume) for the two rice varieties.

Figure 7 provides a visual validation of the optimization process through overlaid contour plots, which define the operability region corresponding to optimal predicted responses. The superimposition of contour plots for all response variables (hardness, springiness, chewiness, moisture, and specific volume) allowed the identification of formulation domains satisfying multiple criteria simultaneously. The optimal region for red rice formulations was located at high water content (approximately 100 mL) and low BCSM levels (0–1 g), indicating that increased hydration is required to compensate for the structural rigidity of red rice flour. Conversely, the optimal region for brown rice formulations shifted toward higher BCSM levels (around 10 g) and lower water content (approximately 77 mL), suggesting that the brown rice matrix achieves optimal performance at higher solid content and reduced hydration levels.

### 3.5. Comparison of the Characteristics of Control and Optimized Gluten-Free Bread

#### 3.5.1. Dynamic Rheological Characteristics of the Bread Dough

**Figure 8:**
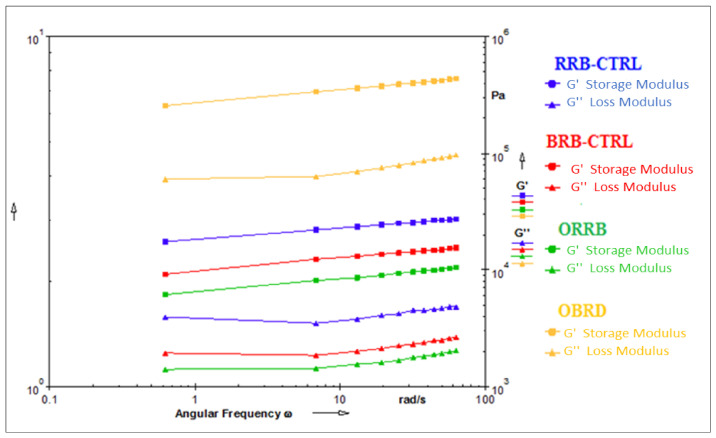
Frequency dependence of storage modulus (G′) and loss modulus (G″) of gluten-free bread dough formulations (RRB-CTRL, BRB-CTRL, ORRB, and OBRB).

The storage modulus (G′) represents the elastic component of the dough, reflecting its stiffness and ability to store energy in a deformable form, whereas the loss modulus (G″) describes the viscous component, indicating energy dissipation as heat. The damping factor (tan δ = G″/G′) provides insight into the viscous-to-elastic ratio of the system. A low tan δ indicates dominant elastic (solid-like) behavior, while a higher value reflects a more viscous (liquid-like) character. In particular, tan δ < 1 denotes a solid-like viscoelastic behavior, which is essential for maintaining structural integrity in baking systems. As shown in Figure 8, both G′ and G″ increased monotonically with angular frequency for all samples, a behavior typical of viscoelastic solid materials in which stiffness increases with deformation rate [38]. Across the entire frequency range, G′ consistently exceeded G″, confirming that all dough systems exhibited predominantly elastic behavior.

This characteristic is particularly relevant in gluten-free baking, as it ensures the presence of a structured network capable of retaining carbon dioxide produced during fermentation. Among the tested formulations, the optimized brown rice bread (OBRB) dough exhibited markedly higher G′ and G″ values compared to the other samples (BRB-CTRL, RRB-CTRL, and ORRB). In particular, G′ values were more than ten times higher, indicating the formation of a highly dense and deformation-resistant molecular network. This pronounced effect can be attributed to the high incorporation level of black cumin seed meal (10 g), which acts as a structuring agent. In agreement with [14], black cumin by-products can promote gel formation and enhance viscoelastic structuring in complex food matrices. Moreover, the relatively low slope of the G′ curves as a function of angular frequency suggests excellent mechanical stability, indicating that the network structure remains intact even under increasing mechanical stress. Although OBRB exhibited the highest G′ and G″ values, indicating the strongest and most structured viscoelastic network, it did not necessarily present the lowest tanδ value. This apparent discrepancy can be explained by the fact that tanδ reflects the ratio between viscous and elastic components rather than their absolute magnitude. Therefore, the simultaneous increase in both G′ and G″ in OBRB resulted in a highly structured dough system while maintaining a relatively balanced viscoelastic behavior.

Regarding the control samples, the red rice control (RRB-CTRL) exhibited a slightly stronger structure than the brown rice control (BRB-CTRL), despite its similar proximate composition. This suggests that red rice flour may promote stronger intermolecular interactions, possibly due to its longer amylopectin branch chains compared to those of brown rice.

The OBRB formulation showed the highest modulus values (G′ ranging from 255,000 to 434,000 Pa, +70%; G″ from 59,900 to 96,100 Pa), confirming its superior stiffness. This behavior can be explained by the combined effect of high BCSM content (10 g) and reduced water content (77 mL). The fiber- and protein-rich BCSM reinforces the network, while the reduced water content increases polymer concentration (starch and proteins), resulting in a compact and highly resistant structure. According to [39], the strong water-binding capacity of black cumin flour contributes to mimicking gluten functionality by limiting free water and enhancing dough viscosity. Additionally, hydrated proteins from black cumin may interact with rice proteins through hydrogen bonding and disulfide interactions, further strengthening the network. The fibrous fraction of *Nigella sativa*, rich in non-starch polysaccharides such as glucose, rhamnose, xylose, and arabinose, contributes to the formation of mucilaginous structures [40], which further enhance matrix stiffness (G′). In contrast, the ORRB formulation (characterized by low BCSM content, presence of whey, and high water level) exhibited the lowest modulus values (G′: 6150–10,500 Pa; G″: 1410–2040 Pa), indicating a softer and less structured dough. The high water content (100 mL) acts as a plasticizer, reducing intermolecular interactions and decreasing network density. Although whey proteins contribute to network formation, their effect was insufficient to counterbalance the dilution induced by the high hydration level.

**Figure 9:**
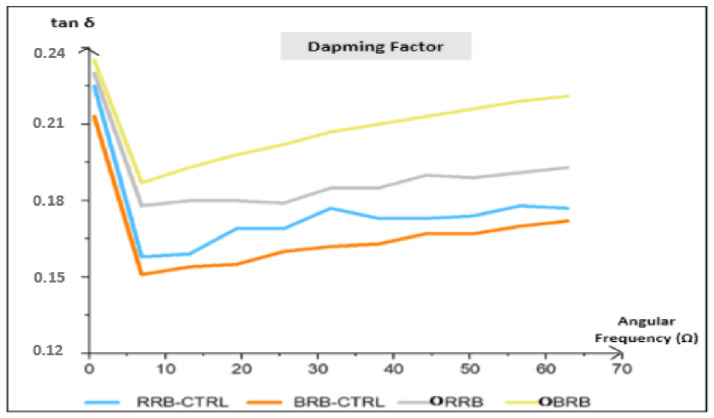
Frequency dependence of the damping factor (tan δ) of gluten-free bread dough formulations (RRB-CTRL, BRB-CTRL, ORRB, and OBRB).

The analysis of the damping factor (tan δ) further supports these findings. Values ranged between 0.15 and 0.24, confirming the dominance of elastic behavior in all samples. Lower tan δ values indicate stronger network structures. As shown in Figure 9, tan δ slightly decreased with increasing angular frequency, indicating a progressive shift toward more elastic behavior under higher deformation rates, which is advantageous during processing stages such as mixing and baking. The limited variation in tanδ across frequencies suggests that all dough systems behave as weak gels, a typical characteristic of gluten-free matrices structured by fibers [41,42].

#### 3.5.2. Physicochemical Characteristics of Optimized Gluten-Free Bread

Table 7 presents the physicochemical characteristics of the optimized gluten-free bread formulations (OBRB and ORRB) and their respective control samples. The results for specific volume (Vsp) indicate no statistically significant differences between the optimized formulations (*p* > 0.05). The values ranged from 2.21 to 2.39 cm^3^/g for OBRB and ORRB, respectively, suggesting relatively compact crumb structures with limited gas retention. Specific volume is a primary indicator of bread quality, reflecting the ability of the dough matrix to retain gas during fermentation and baking. Although the ORRB formulation, enriched with whey, exhibited slightly higher mean values than OBRB, the difference was not statistically significant (*p* = 0.25). These findings are consistent with [20], who reported that whey protein incorporation can enhance the specific volume of gluten-free bread. A higher specific volume is generally desirable, as it is strongly associated with consumer perception of bread quality [43].

Weight loss during baking, mainly attributed to water evaporation, showed no significant differences among samples (*p* = 0.43), with values ranging between 23% and 24%. This consistency suggests that the water-binding capacity of the ingredients used in both control and enriched formulations was comparable. Therefore, the incorporation of BCSM and whey did not significantly alter moisture migration or thermal behavior during baking. Since moisture content is a critical factor influencing bread shelf-life, these results indicate that the optimized formulations maintain stability comparable to conventional gluten-free systems.

Regarding pH, highly significant differences were observed (*p* < 0.0001). Control samples (BRB-CTRL: 6.36; RRB-CTRL: 6.49) exhibited higher pH values compared to the enriched breads (OBRB: 6.16; ORRB: 6.21). This reduction suggests that the incorporation of *Nigella sativa* seed meal and whey contributes to slight acidification of the dough. Similar trends have been reported in studies involving whole-grain pigmented rice and functional fiber incorporation, where bioactive compounds such as phenolic acids influence fermentation dynamics and final pH. A pH range of 6.1–6.2 is generally considered favorable for crumb elasticity and flavor development. In agreement, Awulachew [17] reported a significant pH decrease from 5.92 in control bread to 5.60 in black cumin-enriched formulations.

Water activity (aw) did not differ significantly among samples (*p* = 0.94), remaining within the range of 0.84–0.85. This indicates that the enrichment strategy did not affect the availability of free water, a key parameter for microbial stability. Maintaining aw below 0.90 is essential to prevent rapid mold growth in gluten-free products. Therefore, the observed values confirm that the optimized formulations successfully balance moisture retention and shelf-life stability.

Color analysis revealed highly significant differences (*p* < 0.0001) for both crust and crumb parameters. The inclusion of functional ingredients markedly influenced the chromatic properties of the bread samples. In particular, the OBRB formulation exhibited a pronounced reduction in lightness (L*) for both crust (36.90) and crumb (44.37) compared to the control. This darkening effect is primarily attributed to the natural pigmentation and phenolic composition of *Nigella sativa* seed meal, in agreement with [44]. Similar observations were reported by [17], who demonstrated that crumb lightness decreases with increasing levels of black cumin flour.

In terms of crumb color coordinates, the ORRB sample exhibited significantly higher redness (a* = 6.32) compared to OBRB (a* = 0.87), reflecting the intrinsic pigmentation of red rice. Additionally, OBRB showed a significant reduction in yellowness (b*) relative to the control, indicating a shift toward a darker and more neutral color profile. These findings are consistent with recent studies on pigmented rice flours, which highlight their role as natural colorants in gluten-free bakery products. While conventional gluten-free bread is often criticized for its pale appearance, the use of red or brown rice enhances visual appeal, producing a whole-grain-like appearance that is increasingly valued by consumers seeking clean-label and rustic products.

**Figure 10:**
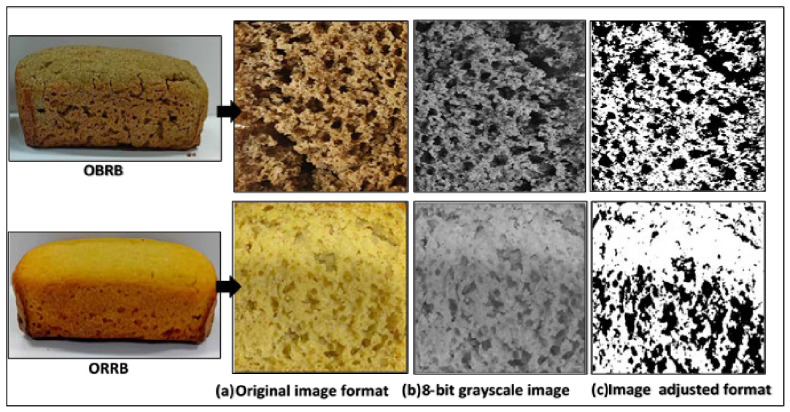
Crumb cell structure analysis of gluten-free bread samples (OBRB and ORRB) using ImageJ software: (**a**) crumb structure image, (**b**) grayscale image, and (**c**) binarized image used for pore quantification.

Crumb structure parameters exhibited lower statistical variability compared to color attributes. Specifically, the number of cells per mm^2^ (*p* = 0.10) and average cell size (*p* = 0.60) showed no significant differences, indicating that the incorporation of functional ingredients did not adversely affect gas cell formation or retention. However, significant differences were observed for perimeter (*p* = 0.02) and circularity (*p* = 0.04). Notably, the ORRB sample achieved the highest circularity value (0.84), suggesting a more uniform and well-developed pore structure (Figure 10).

To summarize, despite the dense fiber network, OBRB maintained high processing stability. Its specific volume (2.21 cm^3^/g), baking weight loss (23%), water activity (0.84), and gas cell micro-structure (pore circularity and density) remained statistically comparable (*p* > 0.05) to the control baseline, ensuring excellent gas retention and microbial shelf-life. A favorable crumb acidification was also achieved (pH 6.16).

#### 3.5.3. Proximate Biochemical Characteristics of the Gluten-Free Bread Formulations

The biochemical characteristics of the gluten-free bread formulations, including Optimized Brown Rice Bread (OBRB), Optimized Red Rice Bread (ORRB), and their respective controls (CTRL), are summarized in Table 8. The primary observation is that, while most macronutrients remained relatively stable, the incorporation of functional ingredients resulted in a highly significant increase in dietary fiber content. Statistical analysis revealed that protein, fat, moisture, ash, and carbohydrate contents did not differ significantly among the formulations (*p* > 0.05). Although the OBRB sample showed a slight numerical increase in protein content (6.70%) compared to its control (6.32%), this difference was not statistically significant (*p* = 0.07), suggesting that the substitution strategy preserved the fundamental nutritional profile of the base formulation. Despite the lack of statistical significance, protein content remains a key parameter in gluten-free bread systems, as it contributes to both structural properties and nutritional value. In this context, even moderate increases may positively influence crumb structure and overall product quality, as reported in previous studies.

Moisture content, although not significantly different, exhibited slightly higher values in the OBRB formulation. This increase may contribute to a softer crumb structure but could also influence shelf-life stability. This observation is consistent with the findings of [45], who highlighted the water-holding capacity of black cumin press cake as a critical functional property. This characteristic plays a central role in water-binding mechanisms within food matrices, thereby modulating key quality parameters such as texture, flavor retention, and shelf life. Similarly, Kamandloo et al. [46] reported that the incorporation of 3–9% BCSM in muffin formulations enhanced moisture retention during storage, indicating that fiber-rich components act as effective water-binding agents within the crumb matrix.

Although ash content (representing total mineral content) did not show statistically significant differences (*p* = 0.20), ORRB exhibited a higher numerical value (4.61%) compared to its control (3.25%). This trend may be attributed to the naturally higher mineral content typically associated with pigmented rice varieties, particularly red rice, compared to refined starch-based systems. Consistent with the stability observed for carbohydrates and lipids, the overall caloric value remained relatively constant across all samples, ranging from approximately 195.00 to 201.57 kcal/100 g, indicating that formulation modifications did not substantially alter the energetic profile of the products. The most relevant finding of this analysis is the highly significant increase in dietary fiber content (*p* = 0.0006). Both enriched formulations (OBRB and ORRB) showed a marked improvement compared to their respective controls, with OBRB reaching the highest fiber level (1.65 ± 0.01%). These results are in agreement with previous studies by [15,17], which demonstrated that the incorporation of black cumin-derived ingredients significantly enhances the nutritional profile of bakery products, particularly through fiber enrichment.

#### 3.5.4. Texture Profile Analysis of Gluten-Free Bread

The results of the texture profile analysis (TPA) of gluten-free bread samples indicate that BRB-CTRL and OBRB exhibited significantly lower hardness values compared to RRB-CTRL and ORRB (*p* = 0.0003) (Table 9). In contrast, chewiness and springiness did not show statistically significant differences among the formulations (*p* > 0.05).

Hardness is a key parameter reflecting crumb firmness and represents one of the main technological challenges in gluten-free bread due to the absence of a gluten network. In many gluten-free systems, hardness values can exceed 1000 g, often correlating with rapid staling and poor consumer acceptability. In this context, the lower hardness observed in OBRB suggests a clear improvement in textural quality.

The results indicate that the incorporation of *Nigella sativa* seed meal plays a functional role in modulating crumb structure. Specifically, BCSM appears to interfere with starch retrogradation and/or act as a plasticizing agent within the matrix, thereby reducing crumb firmness without significantly affecting elastic properties or mastication perception.

These findings are consistent with previous studies. Lower hardness values associated with fiber incorporation have been reported by [47,48], where dietary fibers were shown to reduce crumb rigidity in gluten-free formulations.

More recently, Yüksel et al. [49] demonstrated that fiber-rich ingredients such as *Nigella sativa* seed meal enhance water retention in gluten-free bread systems, contributing to reduced initial hardness. Similarly, Rózyło et al. [39] reported that the incorporation of black cumin seeds and their pressing by-products significantly attenuated key textural parameters, including hardness, elasticity, and chewiness in starch-based breads.

Overall, these results confirm that the strategic use of agro-industrial by-products rich in dietary fiber can effectively mitigate one of the major technological limitations of gluten-free bread, improving texture while maintaining structural integrity.

#### 3.5.5. Sensory Evaluation

The sensory attributes of the experimental gluten-free bread formulations are summarized in Table 10. Statistical analysis revealed no significant differences (*p* > 0.05) across all evaluated parameters among the four samples (BRB-CTRL, OBRB, RRB-CTRL, and ORRB).

These results indicate that the incorporation of *Nigella sativa* seed meal, whey, and the use of different rice flours did not adversely affect the overall sensory profile of the bread. Overall acceptability scores ranged from 5.76 to 6.22, reflecting a neutral-to-positive perception by the panelists. This outcome is particularly relevant, as gluten-free products are often associated with inferior sensory quality compared to wheat-based counterparts. The present findings are consistent with previous studies reporting that plant-based or bran-derived ingredients can be successfully incorporated into gluten-free matrices without compromising consumer acceptance [39].

The absence of significant differences in taste and odor suggests that the functional ingredients used in the OBRB and ORRB formulations did not introduce off-flavors or undesirable aromatic compounds. This is in agreement with the work of [20] who demonstrated that the incorporation of whey into sorghum–rice gluten-free bread preserves sensory integrity while maintaining product quality.

Texture is typically one of the most critical and challenging attributes in gluten-free baking due to the absence of a gluten network. In the present study, although texture scores for RRB-CTRL (4.06 ± 2.61) and BRB-CTRL (5.68 ± 2.87) were numerically lower than those of the enriched formulations, these differences were not statistically significant (*p* > 0.05). Similarly, alveolation (crumb porosity) remained comparable across all samples, indicating that the structural organization of the crumb was not negatively affected by the addition of functional ingredients.

This stability can be attributed to the synergistic role of the incorporated by-products in maintaining dough rheology and crumb structure. In particular, the functional properties of black cumin seed meal, including its emulsifying capacity and its protein–fiber matrix, contribute to structural reinforcement, as reported by [14]. These findings are further supported by [49], who highlighted that cold-pressed seed meals can enhance nutritional value while preserving rheological stability in bread systems.

Within gluten-free formulations, rice flour appears to play a key role in modulating the sensory impact of black cumin. Specifically, rice-based matrices may mitigate the intense flavor profile typically associated with *Nigella sativa*, resulting in improved overall acceptability compared to starch-based systems. Moreover, the combination of dietary fiber and proteins from the cumin meal contributes to a reinforced crumb structure, which positively influences perceived texture and alveolation.

According to [17], the sensory profile of bread is strongly influenced by the concentration of black cumin, with higher inclusion levels leading to decreased acceptability due to increased bitterness, darker color, and stronger aroma. These constraints highlight the importance of optimizing inclusion levels. Similarly, Karaoğlan [14] emphasized that formulation adjustments are necessary to balance functional benefits with sensory quality, particularly considering the high water-binding capacity of black cumin-derived ingredients.

Finally, Zarringhalami et al. [9] reported that the incorporation of Roselle seed and egg white powders significantly influenced hardness, porosity, and sensory attributes of gluten-free bread, whereas hydrocolloids such as xanthan gum showed limited impact on sensory perception. In line with these findings, the present study demonstrates that carefully selected functional ingredients can improve nutritional and structural properties without compromising sensory acceptance. Although relatively high standard deviations were observed for some sensory attributes, particularly taste and aroma, no significant differences (*p* > 0.05) were detected among formulations according to one-way ANOVA followed by Tukey’s HSD test. The observed variability reflects the intrinsic differences in individual sensory perception and preference among panelists.

#### 3.5.6. Analysis of Antioxidant Activity (DPPH Assay)

**Figure 11:**
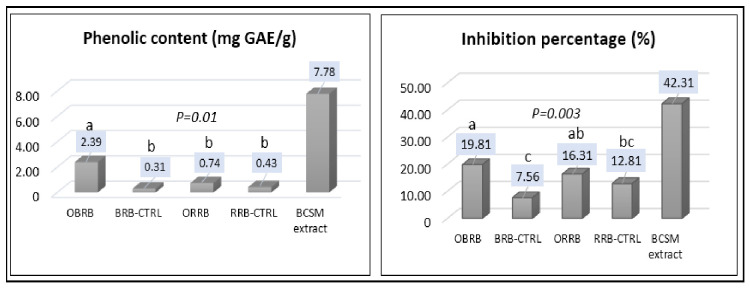
Total phenolic content (TPC, mg GAE/g) and DPPH radical scavenging activity (inhibition percentage, PI%) of gluten-free bread formulations (OBRB, ORRB) and their corresponding controls (BRB-CTRL, RRB-CTRL), compared with the BCSM extract. Different letters indicate significant differences (*p* < 0.05).

The experimental results demonstrate a clear positive correlation between total phenolic content (TPC, mg GAE/g) and antioxidant activity, expressed as percentage inhibition (PI%). As illustrated in Figure 11, the raw BCSM extract exhibited significantly superior performance compared to all other samples, showing the highest phenolic content (7.78 mg GAE/g) and the highest inhibition percentage (42.31%). The total phenolic content observed in the present study exceeds the values reported by [50], who found 26.44 ± 0.21 μg GAE/g extract in *Nigella sativa* oil extracts. In a related context, Hadiqa et al. [51] demonstrated that the incorporation of red rice flour into wheat-based bread significantly increased both TPC and DPPH radical scavenging activity compared to control formulations, confirming the role of pigmented cereals as sources of bioactive compounds. Samples with lower phenolic concentrations, such as BRB-CTRL (0.31 mg GAE/g) and RRB-CTRL (0.43 mg GAE/g), consistently exhibited the lowest inhibition percentages (7.56% and 12.81%, respectively). In contrast, the OBRB formulation, enriched with the highest level of BCSM (10 g), exhibited the highest total phenolic content (2.39 mg GAE/g; *p* = 0.01) and a correspondingly superior antioxidant activity (19.81%; *p* = 0.003) compared to the other bread formulations. This clearly confirms that BCSM enrichment is the primary driver of the functional properties of the developed gluten-free bread. These findings are consistent with numerous studies highlighting the strong antioxidant potential of black cumin seed meal, attributed to its rich composition in phenolic compounds and thymoquinone. These bioactive molecules are known to inhibit lipid peroxidation and protect food systems from oxidative degradation [14,15,52]. The HPLC chromatographic profile (Figure 1) provides mechanistic support for the observed antioxidant activity by identifying the key phenolic compounds present in BCSM. The detection of gallic, protocatechuic, syringic, and ellagic acids, together with hydroxycinnamic acids such as caffeic, *p*-coumaric, and ferulic acids, suggests a multi-level antioxidant defense system. Additionally, the presence of flavonoids such as quercetin, myricetin, and kaempferol is particularly relevant, as these compounds are well known for their strong radical-scavenging capacity. Interestingly, the slightly higher antioxidant activity observed in OBRB compared to ORRB suggests a potential synergistic interaction between the phenolic compounds of brown rice fibers and those derived from BCSM. This synergy may enhance the overall antioxidant efficiency of the matrix, highlighting the importance of ingredient selection and interaction in the design of functional gluten-free products.

## 4. Conclusions

In summary, the multi-response optimization framework successfully identified the Optimized Brown Rice Bread (OBRB) formulated with 10 g of black cumin seed meal (BCSM) and a reduced hydration level of 77 mL as the superior matrix. This optimized formulation effectively resolved the traditional quality bottlenecks inherent to gluten-free breadmaking. Crucially, the fiber-rich BCSM fractions acted as natural hydrocolloids and plasticizing agents, which mitigated crumb hardness and hindered starch retrogradation kinetics during storage without compromising crumb springiness.

Furthermore, the formulation-dependent incorporation of BCSM and whey, particularly in the brown rice matrix, improved the nutritional and phytochemical profile of the optimized products compared with the corresponding control breads. This functional fortification was achieved without compromising key technological and physicochemical parameters, including specific volume, bake loss, or water activity. While textural profiles, specifically crumb firmness, were markedly improved by the dietary fiber and whey protein network, the bread maintained its structural integrity and optimal crumb moisture. These findings demonstrate that the valorization of agro-industrial by-products offers a sustainable and technologically viable pathway to overcome the structural defects of gluten-free matrices, yielding products that are simultaneously nutritionally dense, texturally stable, and commercially promising. The optimized red rice bread formulation consisted of 0.4 g BCSM, 5.02 g whey powder, 100 mL dough water, and 5 mL pre-hydration water, whereas the optimized brown rice bread formulation consisted of 10.0 g BCSM, 70 mL dough water, and 32 mL pre-hydration water. Overall, the results indicate that BCSM and whey can be used as complementary, formulation-dependent ingredients rather than necessarily requiring simultaneous high-level incorporation. In the brown rice matrix, BCSM was the primary driver of functional enrichment, contributing to increased dietary fiber, enhanced antioxidant potential, and improved crumb softness. Conversely, in the red rice matrix, whey played a more prominent role in promoting loaf expansion and structural development, while only a low amount of BCSM was required to maintain formulation balance. Therefore, the optimized formulations highlight two distinct technological strategies for gluten-free bread improvement: BCSM-driven functional enhancement in OBRB and whey-assisted structural optimization in ORRB.

## Figures and Tables

**Table 1:** Experimental matrix of the 40 trials for gluten-free bread formulation based on a Central Composite Design (CCD) with four factors (water, whey, BCSM, and rice variety).

Trial	Coded Values				Uncoded Values			
	X_1_ (Water, mL)	X_2_ (Whey, g)	X_3_ (BCSM, g)	X_4_ (Rice)	X_1_ (Water, mL)	X_2_ (Whey, g)	X_3_ (BCSM, g)	X_4_ (Rice)
1	0	1.68	0	RR	88.50	7.00	5.00	RR
2	0	1.68	0	BR	88.50	7.00	5.00	BR
3	−1	1	1	RR	81.66	5.581	7.97	RR
4	1	1	1	BR	95.33	5.58	7.97	BR
5	1.68	0	0	RR	100	3.50	5.00	RR
6	−1	1	−1	RR	81.66	5.58	2.02	RR
7	0	0	0	RR	88.50	3.50	5.00	RR
8	−1	−1	1	BR	81.66	1.41	7.97	BR
9	1	1	1	RR	95.33	5.58	7.97	RR
10	−1	−1	−1	BR	81.66	1.41	2.02	BR
11	−1	1	−1	BR	81.66	5.58	2.02	BR
12	−1	−1	1	RR	81.66	1.41	7.97	RR
13	0	0	−1.68	BR	88.50	3.50	0.00	BR
14	0	0	−1.68	RR	88.50	3.50	0.00	RR
15	1	−1	1	BR	95.33	1.41	7.97	BR
16	1	1	−1	RR	95.33	5.58	2.02	RR
17	0	0	0	BR	88.50	3.50	5.00	BR
18	0	0	0	RR	88.50	3.50	5.00	RR
19	−1	−1	−1	RR	81.66	1.41	2.02	RR
20	0	0	0	BR	88.50	3.50	5.00	BR
21	0	0	0	RR	88.50	3.50	5.00	RR
22	0	0	0	BR	88.50	3.50	5.00	BR
23	0	0	0	BR	88.50	3.50	5.00	BR
24	0	0	0	RR	88.50	3.50	5.00	RR
25	1	−1	−1	RR	95.33	1.41	2.02	RR
26	0	0	1.68	RR	88.50	3.50	10	RR
27	1	1	−1	BR	95.33	5.58	2.02	BR
28	1	−1	−1	BR	95.33	1.41	2.02	BR
29	−1.68	0	0	BR	77.00	3.50	5.00	BR
30	0	−1.68	0	BR	88.50	0.00	5.00	BR
31	0	0	1.68	BR	88.50	3.50	10	BR
32	0	0	0	BR	88.50	3.50	5.00	BR
33	1.68	0	0	BR	100	3.50	5.00	BR
34	0	−1.68	0	RR	88.50	0.00	5.00	RR
35	−1.68	0	0	RR	77.00	3.50	5.00	RR
36	0	0	0	BR	88.50	3.50	5.00	BR
37	0	0	0	RR	88.50	3.50	5.00	RR
38	−1	1	1	BR	81.66	5.58	7.97	BR
39	1	−1	1	RR	95.33	1.41	7.97	RR
40	0	0	0	RR	88.50	3.50	5.00	RR

BCSM: black cumin seed meal; RR = formulation containing 48 g red rice flour; BR = formulation containing 48 g brown rice flour. X_1_: water content (mL); X_2_: whey content (g); X_3_: black cumin seed meal content (g); X_4_: rice variety (categorical factor: RR = red rice; BR = brown rice).

**Table 2:** Physicochemical and textural properties of 40 gluten-free bread formulations prepared with different levels of water, whey, BCSM, and rice variety.

Run	Water (mL)	Whey (g)	BCSM (g)	Rice	Specific Volume (cm^3^/g)	Moisture (%)	Hardness (g)	Springiness	Chewiness (g)
1	88.50	7.00	5.00	RR	2.38 ± 0.00 ^bcde^	48.58 ± 0.00 ^abcdefg^	962.35 ± 0.00 ^bcdefg^	0.89 ± 0.00 ^fg^	675.48 ± 0.00 ^def^
2	88.50	7.00	5.00	BR	2.21 ± 0.00 ^efghi^	49.11 ± 0.00 ^abcdefg^	1406.48 ± 0.01 ^ab^	1.00 ± 0.00 ^defg^	910.23 ± 0.0 ^bcdef^
3	81.66	5.58	7.97	RR	2.44 ± 0.00 ^abcde^	47.67 ± 0.01 ^defg^	853.37 ± 0.00 ^cdefgh^	0.93 ± 0.00 ^efg^	615.38 ± 0.01 ^def^
4	95.34	5.58	7.97	BR	2.26 ± 0.00 ^defghi^	52.28 ± 0.01 ^abc^	817.50 ± 0.00 ^defgh^	0.95 ± 0.00 ^efg^	623.52 ± 0.01 ^def^
5	100.00	3.50	5.00	RR	2.52 ± 0.00 ^abc^	49.59 ± 0.00 ^abcdefg^	777.26 ± 0.01 ^efgh^	1.28 ± 0.00 ^bc^	663.17 ± 0.00 ^def^
6	81.66	5.58	2.03	RR	2.12 ± 0.00 ^fghij^	46.39 ± 0.01 ^g^	1167.59 ± 0.00 ^bcdef^	0.97 ± 0.00 ^defg^	1038.86 ± 0.01 ^bcd^
7	88.50	3.50	5.00	RR	2.06 ± 0.01 ^ijklm^	50.72 ± 0.00 ^abcdef^	911.63 ± 0.00 ^bcdefgh^	0.91 ± 0.00 ^fg^	702.37 ± 0.00 ^def^
8	81.66	1.42	7.97	BR	2.09 ± 0.00 ^hijklm^	49.48 ± 0.00 ^abcdefg^	787.26 ± 0.01 ^efgh^	0.98 ± 0.00 ^defg^	653.12 ± 0.01 ^def^
9	95.34	5.58	7.97	RR	2.66 ± 0.00 ^a^	49.50 ± 0.00 ^abcdefg^	726.26 ± 0.01 ^efgh^	0.94 ± 0.00 ^efg^	562.01 ± 0.01 ^def^
10	81.66	1.42	2.03	BR	1.88 ± 0.00 ^klm^	48.04 ± 0.01 ^cdefg^	1336.57 ± 0.01 ^abcd^	1.15 ± 0.00 ^bcdef^	1451.60 ± 0.2 ^ab^
11	81.66	5.58	2.03	BR	1.88 ± 0.0 ^jklm^	47.33 ± 0.00 ^efg^	1229.18 ± 0.01 ^abcde^	1.34 ± 0.01 ^ab^	1442.88 ± 0.01 ^ab^
12	81.66	1.42	7.97	RR	2.33 ± 0.0 ^cdefgh^	48.21 ± 0.01 ^bcdefg^	831.31 ± 0.01 ^cdefgh^	0.98 ± 0.01 ^defg^	715.70 ± 0.07 ^def^
13	88.50	3.50	0.00	BR	1.95 ± 0.00 ^ijklm^	49.09 ± 0.00 ^defg^	1197.57 ± 0.00 ^abc^	1.68 ± 0.00 ^a^	1770.79 ± 0.00 ^a^
14	88.50	3.50	0.00	RR	2.21 ± 0.00 ^efghi^	46.16 ± 0.01 ^abcdefg^	1514.62 ± 0.01 ^bcdefg^	1.21 ± 0.00 ^bcde^	1359.24 ± 0.01 ^cdef^
15	95.34	1.42	7.97	BR	2.18 ± 0.00 ^bcdef^	53.09 ± 0.00 ^abcdefg^	531.75 ± 0.00 ^efgh^	1.19 ± 0.00 ^bcdefg^	388.91 ± 0.00 ^def^
16	95.34	5.58	2.03	RR	2.56 ± 0.00 ^abcd^	48.11 ± 0.00 ^abcdefg^	945.33 ± 0.01 ^bcdefgh^	1.01 ± 0.00 ^bcdefg^	826.44 ± 0.01 ^bcdef^
17	88.50	3.50	5.00	BR	1.93 ± 0.00 ^jklm^	50.75 ± 0.00 ^abcdef^	891.57 ± 0.01 ^bcdefgh^	0.98 ± 0.00 ^defg^	765.51 ± 0.00 ^def^
18	88.50	3.50	5.00	RR	2.05 ± 0.00 ^ijklm^	50.58 ± 0.00 ^abcdefg^	874.60 ± 0.07 ^cdefgh^	0.91 ± 0.00 ^fg^	702.37 ± 0.00 ^def^
19	81.66	1.42	2.03	RR	1.84 ± 0.00 ^m^	46.59 ± 0.00 ^fg^	1708.99 ± 0.01 ^a^	0.95 ± 0.00 ^efg^	1336.82 ± 0.01 ^abc^
20	88.50	3.50	5.00	BR	2.07 ± 0.00 ^hijklm^	51.18 ± 0.00 ^abcde^	866.35 ± 0.00 ^cdefgh^	1.24 ± 0.00 ^bcd^	765.51 ± 0.01 ^def^
21	88.50	3.50	5.00	RR	2.09 ± 0.00 ^hijkl^	51.12 ± 0.00 ^abcde^	911.63 ± 0.00 ^bcdefgh^	0.91 ± 0.00 ^fg^	765.51 ± 0.01 ^def^
22	88.50	3.50	5.00	BR	1.90 ± 0.07 ^lm^	51.01 ± 0.20 ^abcde^	891.57 ± 0.01 ^bcdefgh^	1.24 ± 0.00 ^bcd^	831.77 ± 0.01 ^cdef^
23	88.50	3.50	5.00	BR	1.94 ± 0.00 ^jklm^	51.00 ± 0.70 ^abcde^	874.60 ± 0.07 ^cdefgh^	1.24 ± 0.00 ^bcd^	765.51 ± 0.01 ^def^
24	88.50	3.50	5.00	RR	2.09 ± 0.00 ^hijklm^	50.36 ± 0.00 ^abcdefg^	911.63 ± 0.01 ^bcdefgh^	0.86 ± 0.00 ^g^	797.57 ± 0.01 ^cdef^
25	95.34	1.42	2.03	RR	2.30 ± 0.07 ^bcdefg^	46.58 ± 0.00 ^fg^	1079.03 ± 0.01 ^bcdef^	1.02 ± 0.00 ^cdefg^	1032.32 ± 0.01 ^bcde^
26	88.50	3.50	10.00	RR	2.20 ± 0.07 ^defghi^	50.34 ± 0.00 ^abcdefg^	691.29 ± 0.00 ^fgh^	0.93 ± 0.00 ^efg^	649.03 ± 0.01 ^def^
27	95.34	5.58	2.03	BR	2.04 ± 0.00 ^ijklm^	51.18 ± 0.00 ^abcde^	906.25 ± 0.01 ^bcdefgh^	1.32 ± 0.00 ^ab^	1000.25 ± 0.01 ^def^
28	95.34	1.42	2.03	BR	2.05 ± 0.01 ^ijklm^	52.17 ± 0.00 ^abc^	946.49 ± 0.00 ^bcdefgh^	1.24 ± 0.00 ^bcd^	1039.45 ± 0.00 ^bcd^
29	77.00	3.50	5.00	BR	2.01 ± 0.00 ^ijklm^	48.84 ± 0.01 ^abcdefg^	1003.74 ± 0.00 ^bcdefg^	1.15 ± 0.00 ^bcdef^	927.54 ± 0.01 ^bcdef^
30	88.50	0.00	5.00	BR	2.09 ± 0.00 ^hijklm^	51.61 ± 0.01 ^abcde^	899.64 ± 0.00 ^bcdefgh^	1.11 ± 0.00 ^bcdefg^	679.77 ± 0.01 ^def^
31	88.50	3.50	10.00	BR	2.32 ± 0.00 ^cdefgh^	51.88 ± 0.00 ^abcd^	428.67 ± 0.01 ^h^	1.02 ± 0.00 ^cdefg^	569.57 ± 0.01 ^def^
32	88.50	3.50	5.00	BR	2.00 ± 0.07 ^ijklm^	50.97 ± 0.00 ^abcde^	891.57 ± 0.01 ^bcdefgh^	1.24 ± 0.00 ^bcd^	694.32 ± 0.01 ^def^
33	100.00	3.50	5.00	BR	2.58 ± 0.00 ^ab^	52.67 ± 0.00 ^a^	542.10 ± 0.14 ^gh^	0.96 ± 0.00 ^efg^	429.51 ± 0.01 ^def^
34	88.50	0.00	5.00	RR	2.06 ± 0.00 ^ijklm^	50.06 ± 0.01 ^abcdefg^	944.70 ± 0.14 ^bcdefgh^	0.89 ± 0.00 ^fg^	740.25 ± 0.00 ^def^
35	77.00	3.50	5.00	RR	1.92 ± 0.00 ^jklm^	47.56 ± 0.00 ^defg^	1416.98 ± 0.00 ^ab^	0.94 ± 0.01 ^fg^	1068.21 ± 0.00 ^bcd^
36	88.50	3.50	5.00	BR	2.01 ± 0.00 ^ijklm^	50.88 ± 0.00 ^abcdef^	891.57 ± 0.01 ^bcdefgh^	0.98 ± 0.00 ^defg^	694.32 ± 0.00 ^def^
37	88.50	3.50	5.00	RR	2.06 ± 0.00 ^ijklm^	50.65 ± 0.00 ^abcdefg^	874.60 ± 0.04 ^cdefgh^	0.90 ± 0.00 ^fg^	797.57 ± 0.01 ^cdef^
38	81.66	5.58	7.97	BR	2.11 ± 0.00 ^ghijk^	50.02 ± 0.00 ^abcdefg^	689.54 ± 0.01 ^fgh^	0.98 ± 0.00 ^defg^	489.13 ± 0.03 ^def^
39	95.34	1.42	7.97	RR	2.10 ± 0.02 ^fghijk^	52.48 ± 0.00 ^ab^	856.08 ± 0.00 ^cdefgh^	0.98 ± 0.00 ^defg^	629.10 ± 0.00 ^def^
40	88.50	3.50	5.00	RR	2.08 ± 0.00 ^hijklm^	50.54 ± 0.01 ^abcdefg^	911.63 ± 0.01 ^bcdefgh^	0.90 ± 0.00 ^fg^	768.51 ± 0.01 ^def^
*p*	/	/	/	/	<0.0001	<0.0001	<0.0001	<0.0001	<0.0001

Values are expressed as mean ± standard deviation (*n* = 3). Different lowercase letters within the same column indicate significant differences (*p* < 0.05) according to Tukey’s HSD test. BCSM: black cumin seed meal; RR: red rice = 48 g; BR: brown rice = 48 g.

**Table 3:** Estimated regression coefficients (coded variables) and corresponding *p*-values for each response variable obtained from the central composite design (CCD).

Variable	Coeff.	Vsp (cm^3^/g)	*p*	Moisture (%)	*p*	Hardness	*p*	Chewiness	*p*	Springiness	*p*
Constant	b_0_	2.0233	0.000	50.820	0.000	892.7	0.000	754.6	0.000	1.0249	0.000
X_1_	b_1_	0.1259	0.000	1.154	0.000	−133.5	0.000	−115.7	0.000	0.0236	0.254
X_2_	b_2_	0.0745	0.001	−0.397	0.021	5.1	0.831	−13.5	0.388	−0.0084	0.681
X_3_	b_3_	0.0769	0.001	1.027	0.000	−216.2	0.000	−282.1	0.000	−0.0976	0.000
X_4_	b_4_	0.0645	0.001	−0.769	0.000	46.0	0.026	−11.2	0.389	−0.0912	0.000
X_1_^2^	b_11_	0.0745	0.001	−0.455	0.008	10.1	-	3.8	-	0.0137	-
X_2_^2^	b_22_	0.0480	0.023	−0.393	0.019	51.9	0.033	−3.5	-	−0.0257	-
X_3_^2^	b_33_	0.0436	0.037	−0.561	0.001	18.3	-	115.2	0.000	0.0584	0.006
X_1_X_2_	b_12_	0.0284	-	−0.146	-	44.2	-	30.8	-	−0.0245	-
X_1_X_3_	b_13_	−0.0628	0.026	0.143	-	83.5	0.012	68.9	0.002	0.0001	-
X_1_X_4_	b_14_	0.0137	-	−0.331	-	−15.1	-	17.7	-	0.0281	-
X_2_X_3_	b_23_	0.0147	-	−0.214	-	56.4	-	28.4	-	−0.0391	-
X_2_X_4_	b_24_	0.0528	0.015	0.055	-	−60.3	0.017	−43.6	0.009	0.0022	-
X_3_X_4_	b_34_	−0.0261	-	0.234	-	−4.9	-	69.3	0.000	0.0550	0.012

X_1_: water content; X_2_: whey; X_3_: black cumin seed meal (BCSM); X_4_: rice variety (red rice or brown rice). Coefficients are expressed in coded units. *p*-values < 0.05 indicate statistically significant effects. The *p*-values indicated by a dash show non-significant synergistic and quadratic effects.

**Table 4:** Statistical adequacy parameters of the fitted RSM models.

Response	R^2^ (%)	Adjusted R^2^ (%)	Model *p*-Value	Lack-of-Fit	Lack-of-Fit *p*-Value
Specific volume	81.92	72.88	<0.0001	8.13	0.001
Moisture	86.14	79.21	<0.0001	26.40	<0.0001
Hardness	84.89	77.33	<0.0001	100.52	<0.0001
Chewiness	95.03	92.55	<0.0001	3.97	0.016
Springiness	75.14	62.71	<0.0001	1.36	0.317

R^2^ = coefficient of determination; Adjusted R^2^ = adjusted coefficient of determination; Model *p*-value = significance level of the fitted regression model; Lack-of-fit *p*-value = significance level of the lack-of-fit test.

**Table 5:** Formulation of optimized and control gluten-free bread samples based on brown and red rice.

Ingredient	OBRB	ORRB	BRB-CTRL	RRB-CTRL
Brown rice flour (g)	48.5	0	48.5	0
Red rice flour (g)	0	48.5	0	48.5
Corn flour (g)	48.5	48.5	48.5	48.5
Tapioca starch (g)	3	3	3	3
Black cumin seed meal (g)	10.0	0.4	0	0
Whey powder (g)	0	5.02	0	0
Water for dough (mL)	70	100	100	100
Water for pre-hydration (mL)	32	5	0	0
Salt (g)	2	2	2	2
Yeast (g)	2	2	2	2

OBRB: optimized brown rice bread; ORRB: optimized red rice bread; BRB-CTRL: brown rice bread control; RRB-CTRL: red rice bread control. CTRL samples were prepared without the addition of BCSM and whey.

**Table 6:** Comparison between predicted and experimental values of optimized gluten-free bread formulations.

Values	Sample	Vsp (cm^3^/g)	Moisture (%)	Hardness	Chewiness	Springiness
Predicted values	OBRB	2.59 ^a^	46.98 ^a^	536.79 ^a^	487.24 ^a^	1.05 ^a^
	ORRB	2.85 ^a^	46.08 ^a^	863.53 ^a^	976.83 ^a^	1.26 ^a^
Experimental values	OBRB	2.21 ± 0.14 ^a^	48.12 ± 1.13 ^a^	586.39 ± 112.78 ^a^	546.35 ± 172.96 ^a^	1.11 ± 0.39 ^a^
	ORRB	2.39 ± 0.05 ^a^	47.47 ± 0.81 ^a^	912.53 ± 60.09 ^a^	950.66 ± 231.05 ^a^	1.32 ± 0.68 ^a^

OBRB: optimized brown rice bread; ORRB: optimized red rice bread; Vsp: specific volume. Values are expressed as mean ± standard deviation (*n* = 3). Different lowercase letters within the same column indicate significant differences (*p* < 0.05). No significant differences (*p* > 0.05) were observed between predicted and experimental values according to Student’s *t*-test.

**Table 7:** Physicochemical characteristics of optimized and control gluten-free bread samples.

Parameters	BRB-CTRL	OBRB	RRB-CTRL	ORRB	*p*-Value	Sig.
Vsp (cm^3^/g)	2.16 ± 0.14 ^a^	2.21 ± 0.14 ^a^	2.37 ± 0.08 ^a^	2.39 ± 0.05 ^a^	0.25	ns
Weight loss (%)	23.78 ± 0.89 ^a^	23.19 ± 0.65 ^a^	24.05 ± 0.93 ^a^	23.03 ± 1.30 ^a^	0.43	ns
pH value	6.36 ± 0.01 ^b^	6.16 ± 0.00 ^c^	6.49 ± 0.01 ^a^	6.21 ± 0.00 ^c^	0.0001	***
a_w_	0.84 ± 0.01 ^a^	0.84 ± 0.01 ^a^	0.85 ± 0.04 ^a^	0.84 ± 0.01 ^a^	0.94	ns
Crust color						
L*	55.23 ± 1.70 ^a^	36.90 ± 1.91 ^c^	43.93 ± 1.98 ^b^	40.47 ± 1.19 ^b^	0.0001	***
a*	10.74 ± 1.28 ^c^	5.06 ± 0.97 ^b^	11.11 ± 0.55 ^a^	10.89 ± 0.78 ^a^	0.0001	***
b*	34.24 ± 2.15 ^a^	14.46 ± 1.37 ^c^	20.54 ± 0.74 ^b^	19.09 ± 0.79 ^b^	0.0001	***
Crumb color						
L*	65.72 ± 2.59 ^a^	44.37 ± 0.60 ^d^	48.90 ± 0.94 ^b^	48.43 ± 1.38 ^c^	0.0001	***
a*	−0.67 ± 0.30 ^a^	0.87 ± 0.08 ^b^	6.58 ± 0.33 ^a^	6.32 ± 0.33 ^a^	0.0001	***
b*	28.74 ± 1.41 ^a^	10.80 ± 0.28 ^c^	15.87 ± 0.63 ^b^	15.81 ± 0.62 ^b^	0.0001	***
Crumb structure						
Number of cells/mm^2^	580.50 ± 293.45 ^a^	629.0 ± 76.37 ^a^	385.50 ± 9.19 ^a^	1065.50 ± 276.48 ^a^	0.1	ns
Average cell size (mm^2^)	164.18 ± 80.16 ^a^	176.65 ± 38.99 ^a^	146.52 ± 34.38 ^a^	212.09 ± 10.04 ^a^	0.6	ns
Area fraction (%)	37.15 ± 6.08 ^a^	48.40 ± 3.63 ^a^	48.65 ± 0.67 ^a^	51.64 ± 4.07 ^a^	0.08	ns
Perimeter	39.30 ± 2.74 ^ab^	43.24 ± 2.95 ^ab^	33.74 ± 0.24 ^b^	45.89 ± 2.60 ^a^	0.02	*
Circularity	0.79 ± 0.01 ^b^	0.82 ± 0.00 ^ab^	0.80 ± 0.02 ^ab^	0.84 ± 0.02 ^a^	0.04	*
Solidity	0.85 ± 0.00 ^a^	0.85 ± 0.01 ^a^	0.84 ± 0.01 ^a^	0.87 ± 0.01 ^a^	0.1	ns

OBRB: optimized brown rice bread; ORRB: optimized red rice bread; BRB-CTRL: brown rice bread control; RRB-CTRL: red rice bread control. Vsp: specific volume; aw: water activity. Values are expressed as mean ± standard deviation (*n* = 3). Different lowercase letters within the same row indicate statistically significant differences (*p* < 0.05). ns: not significant; * *p* < 0.05; *** *p* < 0.001.

**Table 8:** Proximate biochemical composition of optimized gluten-free bread formulations and corresponding controls.

Parameters	BRB-CTRL	OBRB	RRB-CTRL	ORRB	*p*-Value	Sig.
Protein (%)	6.32 ± 0.21 ^a^	6.70 ± 0.04 ^a^	6.28 ± 0.08 ^a^	6.28 ± 0.12 ^a^	0.07	ns
Fat (%)	0.75 ± 0.06 ^a^	0.89 ± 0.08 ^a^	0.67 ± 0.04 ^a^	0.80 ± 0.16 ^a^	0.26	ns
Moisture (%)	46.21 ± 0.24 ^a^	48.12 ± 1.13 ^a^	46.51 ± 0.54 ^a^	47.47 ± 0.81 ^a^	0.18	ns
Ash (%)	3.67 ± 0.60 ^a^	3.85 ± 0.28 ^a^	3.25 ± 0.78 ^a^	4.61 ± 0.05 ^a^	0.20	ns
Fiber (%)	1.33 ± 0.03 ^c^	1.65 ± 0.01 ^a^	1.46 ± 0.02 ^b^	1.58 ± 0.02 ^a^	0.0006	***
Carbohydrates (%)	41.72 ± 0.32 ^a^	38.79 ± 0.46 ^a^	41.83 ± 0.48 ^a^	39.26 ± 0.30 ^a^	0.25	ns
Energy (kcal/100 g)	201.57	195.00	200.26	197.36	–	–

BRB-CTRL: Brown rice bread (control); RRB-CTRL: Red rice bread (control); OBRB: Optimized brown rice bread; ORRB: Optimized red rice bread. Values are expressed as mean ± standard deviation (*n* = 3). Different superscript letters within the same row indicate statistically significant differences (*p* < 0.05). Significance levels: *** *p* < 0.001; ns = not significant.

**Table 9:** Texture profile analysis (TPA) parameters of optimized gluten-free bread formulations and corresponding controls.

Parameters	BRB-CTRL	OBRB	RRB-CTRL	ORRB	*p*-Value	Sig.
Hardness (g)	1039.83 ± 227.64 ^a^	586.39 ± 112.78 ^b^	1147.78 ± 128.59 ^a^	912.53 ± 60.09 ^a^	0.0003	***
Chewiness (g)	961.31 ± 320.69 ^a^	546.35 ± 172.96 ^a^	1020.28 ± 312.14 ^a^	950.66 ± 231.05 ^a^	0.50	ns
Springiness	0.98 ± 0.02 ^a^	1.11 ± 0.39 ^a^	1.08 ± 0.36 ^a^	1.32 ± 0.68 ^a^	0.40	ns

BRB-CTRL: Brown rice bread (control); RRB-CTRL: Red rice bread (control); OBRB: Optimized brown rice bread; ORRB: Optimized red rice bread. Values are expressed as mean ± standard deviation (*n* = 3). Different superscript letters within the same row indicate statistically significant differences (*p* < 0.05). Significance levels: *** *p* < 0.001; ns = not significant.

**Table 10:** Sensory evaluation scores of optimized gluten-free bread formulations and corresponding controls.

Parameters	BRB-CTRL	OBRB	RRB-CTRL	ORRB	*p*-Value	Sig.
Taste	5.39 ± 3.03 ^a^	6.02 ± 2.74 ^a^	5.52 ± 2.91 ^a^	6.15 ± 2.49 ^a^	0.90	ns
Color	6.16 ± 2.39 ^a^	6.24 ± 2.24 ^a^	5.70 ± 2.49 ^a^	6.35 ± 2.41 ^a^	0.90	ns
Shape	6.11 ± 2.35 ^a^	6.35 ± 2.49 ^a^	5.95 ± 2.31 ^a^	6.51 ± 2.18 ^a^	0.70	ns
Smell/Odor	5.35 ± 3.09 ^a^	5.69 ± 2.70 ^a^	5.37 ± 2.17 ^a^	5.75 ± 2.21 ^a^	0.90	ns
Texture	5.68 ± 2.87 ^a^	6.42 ± 2.51 ^a^	4.06 ± 2.61 ^a^	5.88 ± 1.93 ^a^	0.10	ns
Alveolation	4.98 ± 1.87 ^a^	5.76 ± 2.59 ^a^	5.90 ± 1.94 ^a^	6.15 ± 2.18 ^a^	0.60	ns
Overall acceptability	5.76 ± 2.04 ^a^	6.04 ± 2.19 ^a^	5.98 ± 2.31 ^a^	6.22 ± 2.19 ^a^	0.90	ns

BRB-CTRL: Brown rice bread (control); RRB-CTRL: Red rice bread (control); OBRB: Optimized brown rice bread; ORRB: Optimized red rice bread. Values are expressed as mean ± standard deviation (*n* = 10). Different superscript letters within the same row indicate statistically significant differences (*p* < 0.05). ns = not significant.

## Data Availability

The original contributions presented in this study are included in the article. Further inquiries can be directed to the corresponding authors.
